# Cortical Composition Hierarchy Driven by Spine Proportion Economical Maximization or Wire Volume Minimization

**DOI:** 10.1371/journal.pcbi.1004532

**Published:** 2015-10-05

**Authors:** Jan Karbowski

**Affiliations:** Institute of Applied Mathematics and Mechanics, University of Warsaw, Warsaw, Poland; Hamburg University, GERMANY

## Abstract

The structure and quantitative composition of the cerebral cortex are interrelated with its computational capacity. Empirical data analyzed here indicate a certain hierarchy in local cortical composition. Specifically, neural wire, i.e., axons and dendrites take each about 1/3 of cortical space, spines and glia/astrocytes occupy each about (1/3)^2^, and capillaries around (1/3)^4^. Moreover, data analysis across species reveals that these fractions are roughly brain size independent, which suggests that they could be in some sense optimal and thus important for brain function. Is there any principle that sets them in this invariant way? This study first builds a model of local circuit in which neural wire, spines, astrocytes, and capillaries are mutually coupled elements and are treated within a single mathematical framework. Next, various forms of wire minimization rule (wire length, surface area, volume, or conduction delays) are analyzed, of which, only minimization of wire volume provides realistic results that are very close to the empirical cortical fractions. As an alternative, a new principle called “spine economy maximization” is proposed and investigated, which is associated with maximization of spine proportion in the cortex per spine size that yields equally good but more robust results. Additionally, a combination of wire cost and spine economy notions is considered as a meta-principle, and it is found that this proposition gives only marginally better results than either pure wire volume minimization or pure spine economy maximization, but only if spine economy component dominates. However, such a combined meta-principle yields much better results than the constraints related solely to minimization of wire length, wire surface area, and conduction delays. Interestingly, the type of spine size distribution also plays a role, and better agreement with the data is achieved for distributions with long tails. In sum, these results suggest that for the efficiency of local circuits wire volume may be more primary variable than wire length or temporal delays, and moreover, the new spine economy principle may be important for brain evolutionary design in a broader context.

## Author Summary

Cerebral cortex is an outer layer of the brain in mammals, and it plays a critical part in various cognitive processes such as learning, memory, attention, language, and consciousness. The cerebral cortex contains a number of neuroanatomical parameters whose values are essentially conserved across species and brain sizes, which suggests that these particular parameters are somehow important for brain efficient functioning. This study shows that the fractional volumes of five major cortical components both neuronal and non-neuronal (axons, dendrites, spines, glia/astrocytes, capillaries) are also approximately conserved across mammals, and neural wire (axons and dendrites) occupies the most of cortical space. Moreover, the fractional volumes form a special hierarchy of dependencies, being approximately equal to integer powers of 1/3. Is there any evolutionary principle of cortical organization that would explain these properties? This study finds that there are two different theoretical principles that can provide answers: one standard related to minimization of neural wire fractional volume, and a new proposition associated with economical maximization of spine content. However, the latter principle produces more robust results, which suggests that spine economical maximization is potentially an alternative to the more common “wire minimization” in explaining the cortical layout. Therefore, the current study can become an important contribution to our understanding (or debating) of the main factors influencing the evolution of local cortical circuits in the brain.

## Introduction

The gray matter of cerebral cortex is composed primarily of neurons with their extended axonal and dendritic processes, synapses (mostly dendritic spines) connecting different neurons, non-neuronal cells called glia (of which astrocytes are likely the most important), and microvasculature (capillaries). It is thought that neurons and synapses are the main computational and functional elements, whereas glia and capillaries serve supporting or modulatory roles associated with supplying metabolic substrates (oxygen and glucose) to neurons and synapses based on their demands [1, 2]. The experimental data show that there are certain scaling regularities in the arrangement of neuronal, synaptic [3–5], and capillary [6] processes. It is commonly hypothesized that these scaling rules may be the consequence of the design principle called neural “wiring minimization” or “wiring economy” [3, 7–10], or efficiency in functionality-cost trade-offs [11–14]. However, there are some indications that wiring length minimization is not enough to explain the pattern of global connections in the macaque cortex and in the nematode *C. elegans* nervous system [15, 16], and even small-scale networks of macaque and cat visual cortices perform sub-optimally in terms of wire length reduction [17]. This opens a possibility for some other optimization principles governing brain architecture.

Here, it is proposed a new, alternative, design principle that can be called “spine economical maximization” or spine economy. It is based on maximization of spine proportion in the cortex with simultaneous penalization of spine size. This scenario is equivalent to requiring that in a given cortical volume there are as many information storing connections between neurons as possible (maximal functionality) that cost as little energy as possible (economical approach, for bigger spines use more energy). Moreover, we require that these connections are efficiently coupled with astrocytes and capillaries, and the whole system of neuronal and non-neuronal elements is treated within a single theoretical framework. The choice of the guiding principle associated with spine economy is motivated by the following empirical results: (i) Axo-spinal synaptic connections are the most numerous in the cortical gray matter [18] and are very important not only for short-term neuronal communication [12], but also for long-term information storage [19]; (ii) Mammalian brain is metabolically expensive [20–22], which means that energy is an important constraint on brain function [14, 23, 24]; (iii) Spines are likely the major users of metabolic energy in the cortical gray matter [25–27], as is reflected in a strong correlation between cortical synaptogenesis and its energetics [27, 28], which suggests that spine number or size can be limited by available energy.

The new hypothetical principle associated with spine economy is tested here for local organization of cortical circuits. Specifically, it is tested if fractional occupancy of space by the main cortical components predicted on a basis of the spine economical maximization principle agrees with empirical data. Fractional distribution of volume taken by neuronal and non-neuronal elements should be an important aspect of local cortical organization, because densities of neurons, glia, and vasculature are mutually correlated across cortical regions and layers [29–31]. Thus, too much space taken by one component can lead, due to competition, to underperformance of other components [32] or to an excessive cortical size [33], both of which can be undesirable for brain efficient design and functionality. Thus, some neuroanatomical balance between fractional volumes of cortical elements seems necessary. Unfortunately, it is virtually not known whether these fractions are variable or preserved across species. This interesting topic was only briefly addressed before, with the suggestion that the combined fraction of neuronal wire (axons and dendrites) can result from minimization of temporal delays in inter-neuronal signaling [8]. However, from an evolutionary perspective, the knowledge of fractional distribution of all major cortical components, also those supplying metabolic energy, should add an important information to our understanding of the geometric layout of the cortex and for testing various hypotheses concerning its design principles [34].

The paper is organized as follows. First, empirical data on fractional volumes of cortical components are analyzed. In particular, we look for regularities in the data within and across species. We build and study a theoretical model of cortical composition with coupled neuronal and non-neuronal elements. Next, we investigate which optimization principle can best explain the empirical facts. Three classes of optimization models are considered. One is based on a standard principle of neural wire minimization and includes minimization of wire length, wire surface area, wire volume, and local temporal delays. Second class is based on a new proposition of spine economical maximization. Third class is a linear combination of the first two types of models, i.e., it mixes wire cost with spine economy. All kinds of models are based on an implicit assumption that evolution had optimized the nervous system according to some rules [13, 14, 35–37].

## Results

### Empirical composition of the cerebral cortex across mammals reveals hierarchical organization

**Fig 1:**
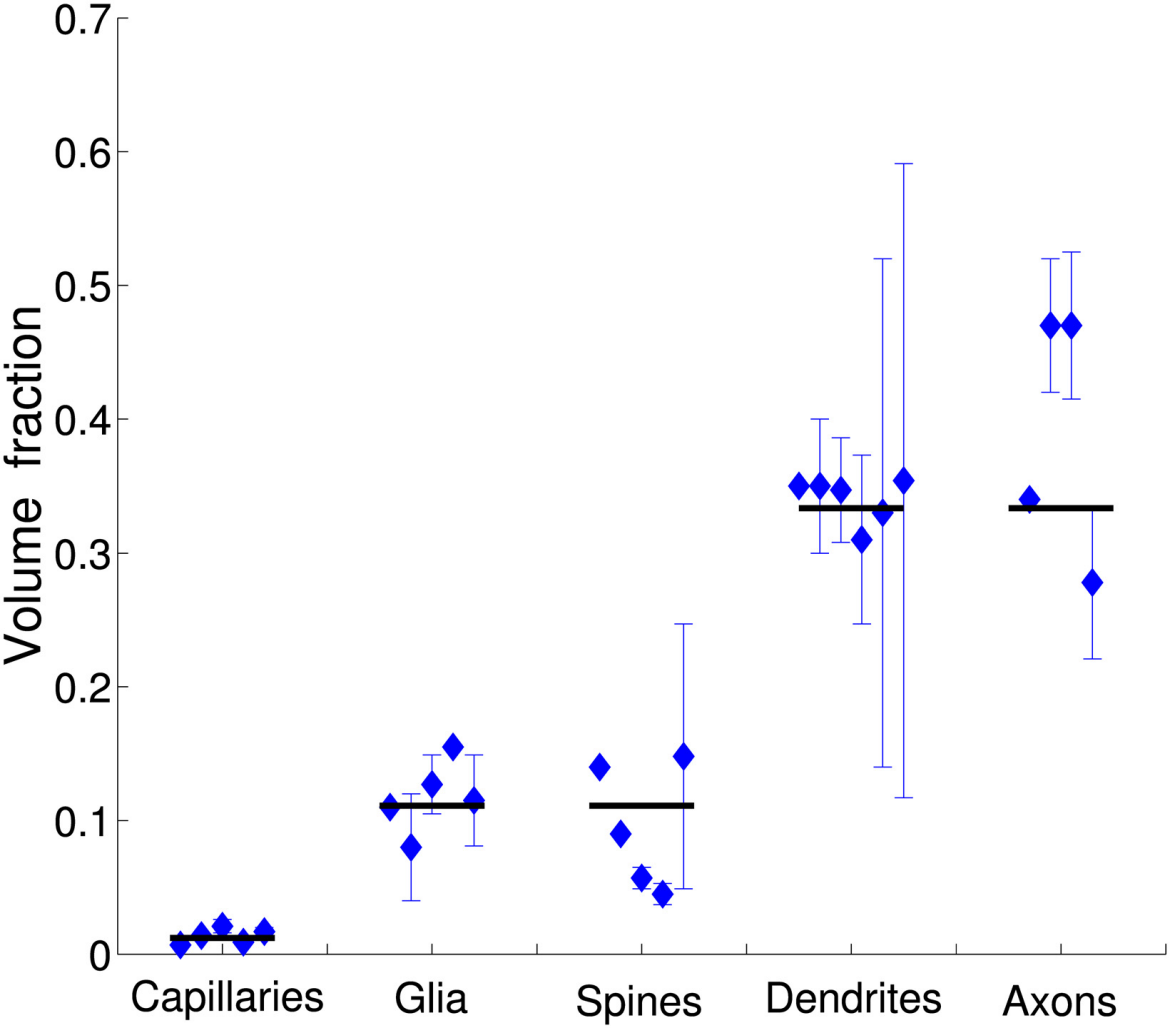
Hierarchical organization of the five major components in the gray matter of cerebral cortex. Empirical data points for each component are denoted by blue diamonds and correspond to different mammals (see Table 1). Solid black lines represent integer powers of the fraction 1/3. In particular, these fractions are: (1/3)^4^ for capillaries, (1/3)^2^ for glia and spines, and 1/3 for dendrites and axons. Note, that on average empirical fractional volumes across species approximately conform to this simple rule.

**Fig 2:**
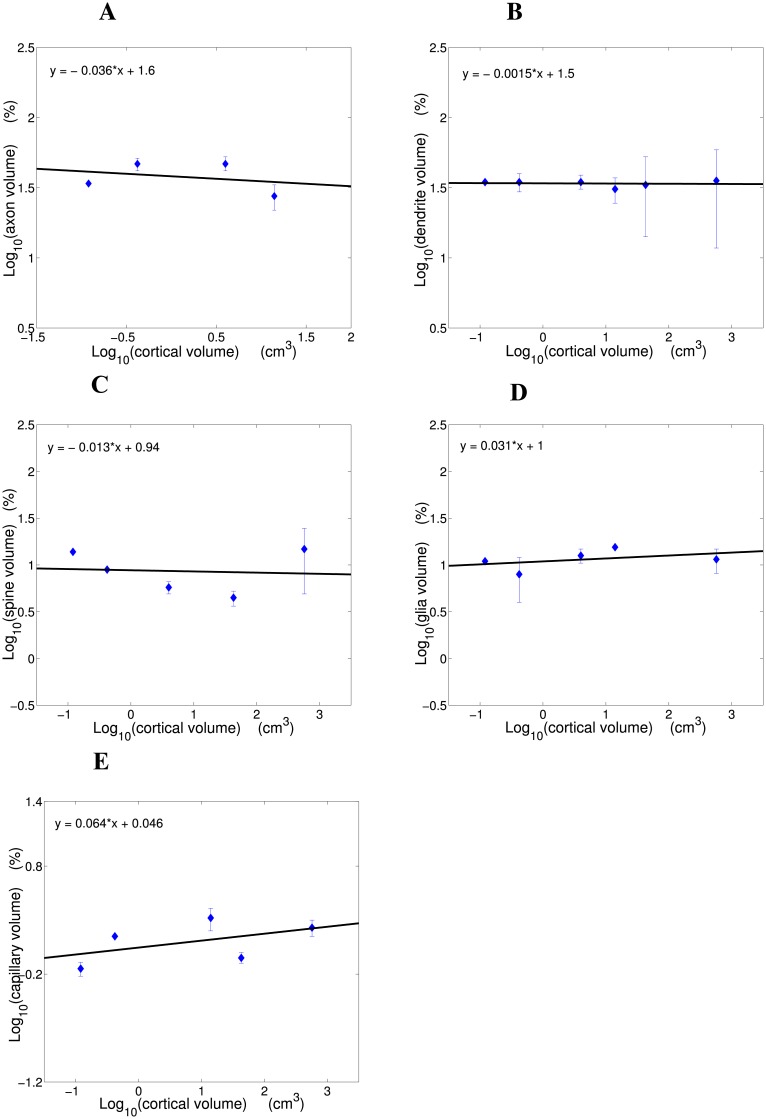
Scaling dependence of fractional volumes of the basic cortical components on cortical gray matter volume. (A) Axon fractional volume, (B) dendrite fractional volume, (C) spine fractional volume, (D) glia fractional volume, and (E) capillary fractional volume as functions of cortical volume in log-log coordinates. Note a conservation trend across mammals. Scaling plots were constructed based on data in Table 1. The following volumes of cortical gray matter (two hemispheres) were used: mouse 0.12 cm^3^ [38], rat 0.42 cm^3^ [39], rabbit 4.0 cm^3^ [40], cat 14.0 cm^3^ [40], macaque monkey 42.9 cm^3^ [39], human 571.8 cm^3^ [39].

Existing experimental data on fractional volumes of cortical gray matter components were analyzed (see the Methods), and it is found that these fractions exhibit a certain hierarchy, since they can be approximated by integer powers of 1/3 (Table 1; Fig 1). Specifically, axons and dendrites occupy each about 1/3 of cortical space, dendritic spines and glia/astrocytes constitute each roughly (1/3)^2^ of the cortex, and capillaries take an extremely small volume fraction around (1/3)^4^ (Table 1; Fig 1). This regularity is called here the rule of “powers of 1/3”. Moreover, an allometric analysis reveals that the fractions of all examined cortical components are species- and brain size independent, i.e. they do not correlate significantly with cortical size and scale with exponents close to zero (Table 1; Fig 2). Typical values for axons: exponent = −0.036, *R* ^2^ = 0.083, *p* = 0.713; for dendrites: exponent = −0.002, *R* ^2^ = 0.037, *p* = 0.717; for spines: exponent = −0.013, *R* ^2^ = 0.008, *p* = 0.885; for glia/astrocytes: exponent = 0.031, *R* ^2^ = 0.180, *p* = 0.477; and for capillaries: exponent = 0.064, *R* ^2^ = 0.243, *p* = 0.399 (Fig 2).

**Table 1:** Structural composition of the gray matter of cerebral cortex.

Species	Volume fraction (%)				
	Axons	Dendrites	Spines	Glia/Astrocytes	Capillaries
Mouse	34.0	35.0	14.0	11.0*	0.7 ± 0.1
Rat	47.0 ± 5.0	35.0 ± 5.0	9.0	8.0 ± 4.0*	1.4
Rabbit	47.0 ± 5.5	34.7 ± 3.9	5.7 ± 0.8	12.7 ± 2.2*	−
Cat	27.8 ± 5.7	31.0 ± 6.3	−	15.5	2.1 ± 0.5
Macaque	−	33.0 ± 19.0	4.5 ± 0.8	−	0.9 ± 0.1
Human	−	35.4 ± 23.7	14.8 ± 9.9	11.5 ± 3.4	1.7 ± 0.3
Species mean	39.0 ± 2.3	34.0 ± 5.3	9.6 ± 2.0	11.7 ± 1.1	1.4 ± 0.1
Normalized mean	40.8 ± 2.4	35.5 ± 5.5	10.0 ± 2.1	12.2 ± 1.2	1.5 ± 0.1
Rule “powers of 1/3”	33.3	33.3	11.1	11.1	1.2

Symbol * corresponds to the fraction of unspecified glia cells. The next-to-last line contains normalized to 100% mean fractional values over species. The last line is a theoretical prediction based on a “powers of 1/3” rule. References for the data are given in the Methods.

### Optimality principles for local cortical circuits

It is possible that the relative constancy of the fractional occupancy of cortical space and its simple hierarchy reflect some kind of evolutionary optimized principle. Three such principles are considered: one associated with neural wire minimization, second with spine proportion economical maximization, and the third proposition is the mixture of the first two. The last choice means that we consider the possibility of some “meta-principle”, which includes the contributions of both primary principles (wire minimization and spine economy maximization) with some weights related to their importance. The most general fitness function *F*, or benefit-cost function, associated with such a meta-principle, which we want to minimize, is given by (see the Methods, in particular the section “The fitness functions” for the derivation)

$$\begin{array}{c} F=f\frac{\left(rx+y\right)}{{\bar{u}}^{\gamma_{1}}}-\left(1-f\right)\frac{s}{{\bar{u}}^{\gamma_{2}}}+\lambda \;(x+y+s+g+c-1), \end{array}$$ (1)

where *x*, *y*, *s*, *g*, *c* are respectively volume fractions of axons, dendrites, spines, glia, and capillaries in the cortical gray matter, and *λ* is the Lagrange multiplier associated with a mathematical constraint of the fractions normalization. The symbol *f* is the control parameter (0 ≤ *f* ≤ 1), or mixing ratio, measuring a relative contribution (importance) to the fitness function of the two contrasting notions: wire minimization and spine economical maximization. If *f* = 1 then the function *F* corresponds to wire cost only, whereas if *f* = 0 then the function *F* describes spine economy only (the negative sign in front of (1 − *f*) is necessary to obtain maximum for spine content). The symbol $\bar{u}$ is the average spine volume, *r* is some positive measure of asymmetry between axons and dendrites, *γ*_1_ and *γ*_2_ are positive exponents. Different values of *γ*_1_ correspond to different kinds of wire minimization. In particular, *γ*_1_ = 0 relates to wire volume minimization, *γ*_1_ = 1/3 corresponds to wire surface area minimization, *γ*_1_ = 2/3 is associated with wire length minimization, and *γ*_1_ = 5/6 relates to local temporal delays minimization (see the Methods). The parameters *r*, *γ*_2_, and *f* constitute the free parameters.

In the next sections we study theoretical consequences of minimization of the fitness function represented by Eq (1). Specifically, we find optimal values of fractional volumes of the cortical components and compare them with the empirical data.

### Optimal fractional volumes of cortical components: Wire minimization vs. spine economical maximization

**Fig 3:**
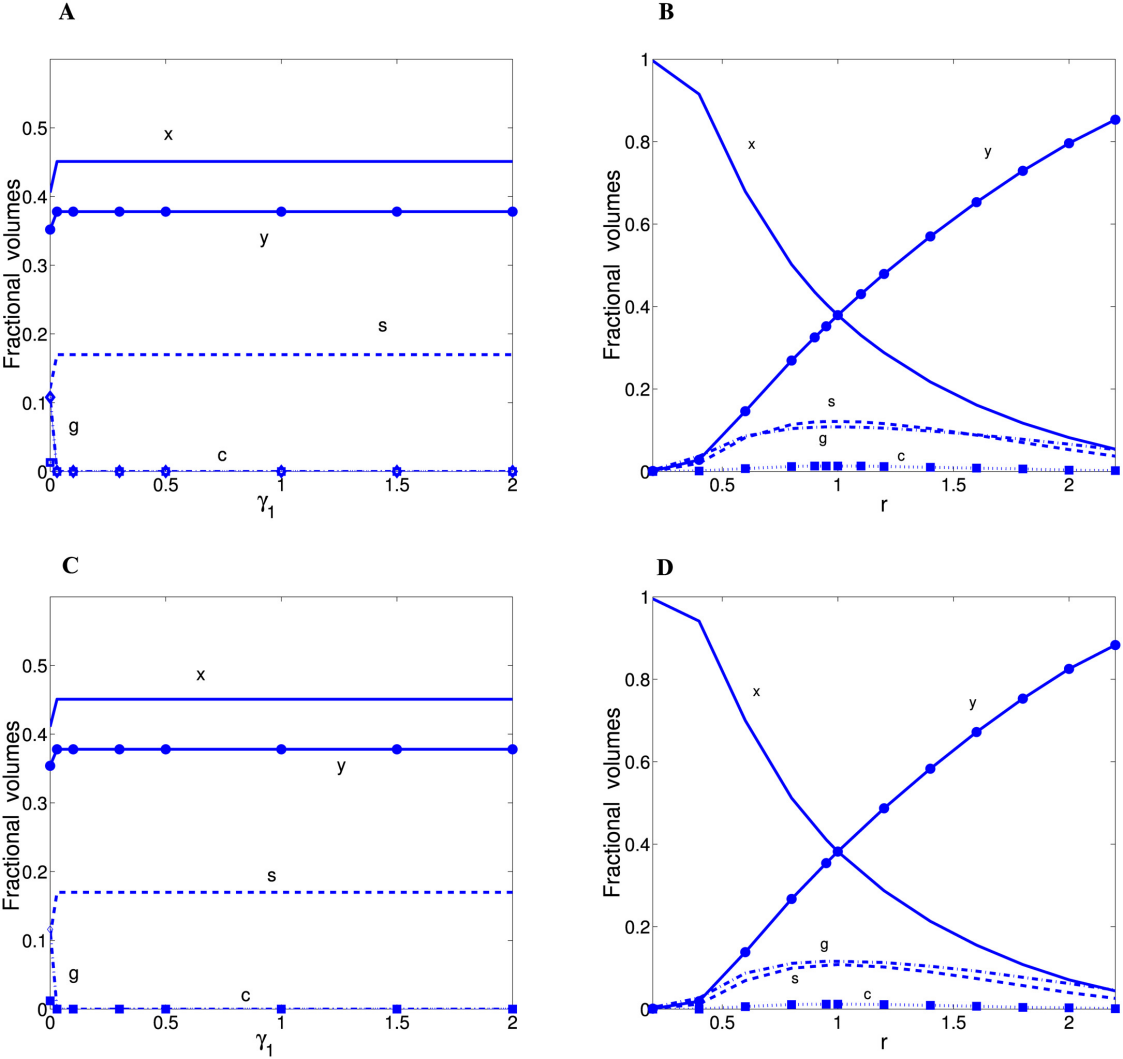
Optimal fractional volumes for neural “wire minimization” principle as functions of the exponent *γ*_1_ and *r*. The results for short-tailed Gamma (n = 2) distribution are in (A) and (B), and for long-tailed Log-normal distribution in (C) and (D). Note that for *γ*_1_ > 0 (panels A and C) all fractions are constant, in particular glia/astrocytes and capillaries vanish (*g* = *c* = 0). Parameter values: for panels (A) and (C) *r* = 0.95, while for panels (B) and (D) *γ*_1_ = 0. Additionally, for log-normal *σ* = 0.3. For all panels *θ* = 0.321 *μ*m^3^.

Wire minimization principle corresponds to *f* = 1 in Eq (1). For this scenario the optimal fractional volumes of cortical components depend on two parameters: *γ*_1_ and *r*. The dependence of optimal fractions on *γ*_1_ is critical. If *γ*_1_ = 0 (the case of wire fractional volume minimization) then the theoretical fractions can be similar to the empirical values in Table 1 (the next-to-last line) only when *r* ∼ 1 (Fig 3). If *γ*_1_ > 0 (the other types of wire minimization), regardless of the value of *r*, the optimal fractions of glia/astrocytes and capillaries vanish, which is unrealistic (Fig 3A and 3C). Moreover, only the case *γ*_1_ = 0 produces finite reliable values of the average spine volume $\bar{u}$ (Table 2); for *γ*_1_ > 0 we obtain $\bar{u}\;\mapsto \;\infty$. The abrupt transition in solutions from *γ*_1_ = 0 to *γ*_1_ > 0 is reminiscent of discontinuous phase transitions. The dependence of the optimal fractional volumes on *r* is more smooth (if *γ*_1_ = 0; Fig 3B and 3D). Realistic fractions are obtained for almost symmetrical situation, i.e. when *r* is close to 1; too small or too large *r* produces vanishing fractions of either dendrites or axons (Fig 3B and 3D). Moreover, all the described results look qualitatively similar regardless of the asymptotic nature of the distributions of spine sizes (compare Fig 3A and 3B vs. Fig 3C and 3D).

**Table 2:** The best optimal theoretical fractional volumes and related parameters in the cortex for a particular case of “wire minimization” principle associated with wire volume minimization (*γ*_1_ = 0). The optimal fractions correspond either to minimal Euclidean distance (ED) or minimal Mahalanobis distance (MD) between theory and data (given in bold face).

Spine size distribution	*θ*	Optimal parameters								ED	MD
		*x*	*y*	*s*	*g*	*c*	$\bar{u}$	*P*	*r*		
Exponential	0.100	0.388	0.330	0.068	0.201	0.014	0.157	0.528	0.94	**0.091**	6.981
	0.100	0.403	0.316	0.067	0.200	0.013	0.157	0.528	0.91	0.094	**6.942**
	0.321	0.423	0.371	0.111	0.085	0.009	0.935	0.709	0.96	**0.045**	6.485
	0.321	0.397	0.397	0.112	0.085	0.010	0.936	0.710	1.00	0.058	**6.360**
Gamma (n = 1)	0.100	0.376	0.316	0.081	0.210	0.017	0.175	0.684	0.93	**0.104**	7.842
	0.100	0.442	0.259	0.078	0.206	0.016	0.175	0.682	0.80	0.134	**7.427**
	0.321	0.413	0.357	0.119	0.099	0.012	0.793	0.805	0.95	**0.030**	3.991
	0.321	0.396	0.374	0.119	0.099	0.012	0.794	0.806	0.98	0.037	**3.892**
Gamma (n = 2)	0.100	0.366	0.310	0.086	0.219	0.019	0.175	0.753	0.93	**0.115**	9.078
	0.100	0.463	0.230	0.080	0.210	0.017	0.174	0.750	0.74	0.163	**8.173**
	0.321	0.406	0.352	0.121	0.108	0.013	0.715	0.846	0.95	**0.026**	2.681
	0.321	0.395	0.363	0.121	0.108	0.013	0.715	0.846	0.97	0.030	**2.640**
Rayleigh	0.100	0.368	0.305	0.082	0.226	0.019	0.159	0.734	0.92	**0.124**	9.623
	0.100	0.471	0.221	0.076	0.216	0.016	0.158	0.731	0.72	0.177	**8.701**
	0.321	0.405	0.352	0.117	0.113	0.013	0.642	0.822	0.95	**0.020**	2.271
	0.321	0.394	0.362	0.117	0.113	0.013	0.642	0.822	0.97	0.025	**2.236**
Log-logistic	0.100	0.404	0.350	0.097	0.136	0.013	0.404	0.683	0.95	**0.015**	2.378
	0.100	0.399	0.356	0.097	0.136	0.013	0.404	0.684	0.96	0.017	**2.351**
	0.321	0.399	0.348	0.124	0.114	0.014	0.671	0.891	0.95	**0.027**	1.803
	0.321	0.403	0.334	0.123	0.124	0.015	0.584	0.910	0.93	0.031	**1.448**
Log-normal	0.100	0.418	0.367	0.072	0.133	0.010	0.311	0.472	0.96	**0.034**	5.734
	0.100	0.401	0.356	0.073	0.158	0.012	0.244	0.513	0.96	0.045	**4.801**
	0.321	0.411	0.354	0.106	0.116	0.012	0.561	0.731	0.95	**0.010**	2.897
	0.321	0.402	0.341	0.110	0.133	0.015	0.475	0.804	0.94	0.021	**1.405**

For log-logistic distribution the minimal ED and MD were obtained for the parameter *β* = 1.5 if *θ* = 0.100, and if *θ* = 0.321 then *β* = 3.5 for minimal ED and *β* = 4.5 for minimal MD. For log-normal distribution the minimal ED was reached for *σ* = 0.7 and minimal MD for *σ* = 0.55 if *θ* = 0.100, and if *θ* = 0.321 then *σ* = 0.3 for minimal ED and *σ* = 0.2 for minimal MD.

**Fig 4:**
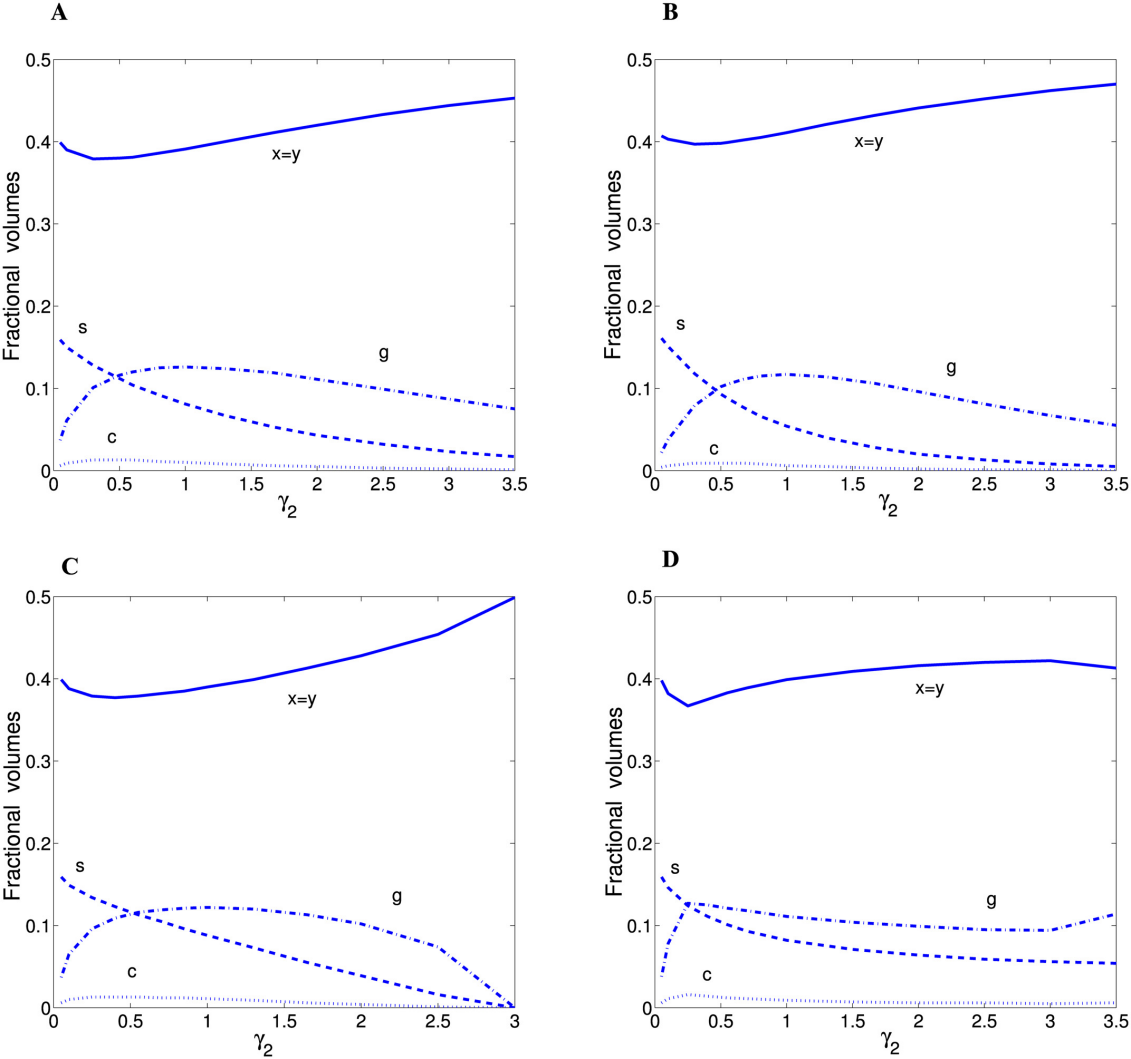
Optimal fractional volumes for “spine economical maximization” principle as functions of the exponent *γ*_2_. The results are qualitatively very similar for (A) Exponential distribution, (B) Gamma (n = 2) distribution, (C) Log-logistic distribution (*β* = 3.0), and (D) Log-normal distribution (*σ* = 0.25). For all distributions *θ* = 0.321 *μ*m^3^.

Alternatively, for spine economical maximization principle, i.e. for *f* = 0 in Eq (1), the optimal fractions of cortical components depend moderately on the exponent *γ*_2_. This dependence is displayed in Fig 4 for four distributions of spine sizes: two with short tails (Gamma and Exponential distributions) and two with long tails (Log-logistic and Log-normal). As can be seen the optimal fractions of axons and dendrites are equal and about 0.4, and they are qualitatively almost independent of *γ*_2_ and spine size distribution. Similarly, the capillary content is relatively stable at about 0.01 or a little less (Fig 4). In contrast, the fractions of spines and astrocytes/glia can change significantly as a function of *γ*_2_. Typically, the spine proportion decreases with increasing *γ*_2_, whereas glia/astrocytes content depends non-monotonically on *γ*_2_, but both of them are restricted from above (spines by ∼ 0.16 and astrocytes by ∼ 0.14). These trends are preserved as we change the distributions of spine volumes from short-range to long-range (Fig 4A and 4B vs. Fig 4C and 4D). The pattern for the remaining two distributions (Gamma n = 1 and Rayleigh; not shown) is very similar. Overall, the optimal fractions of cortical components can either vary with *γ*_2_ or not, but these dependencies are essentially invariant with respect to the distribution type of spine sizes. More importantly, the optimal fractional volumes are quantitatively similar to the empirical values given in Table 1 (the next-to-last line) for a broad range of *γ*_2_.

**Fig 5:**
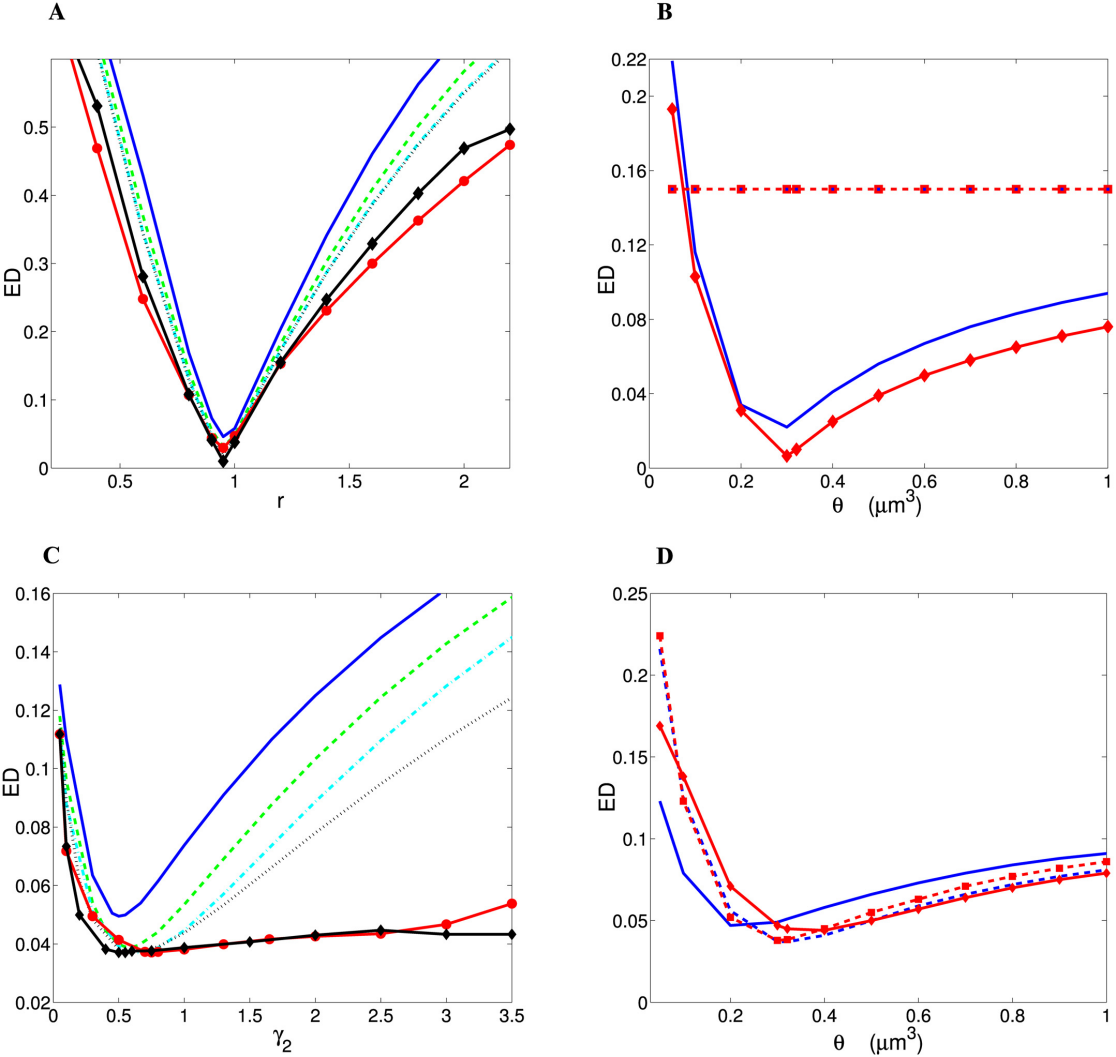
Euclidean distance (ED) between optimal theoretical results and empirical data as a function of *r*, threshold *θ*, and *γ*_1_, *γ*_2_. Panels (A) and (B) refer to “wire minimization”, whereas panels (C) and (D) correspond to “spine economical maximization”. (A) Dependence of ED on *r* for all distributions of spine volumes for “wire volume minimization”, i.e. *γ*_1_ = 0. (B) ED as a function *θ* for *r* = 0.95. Blue lines correspond to Gamma (n = 2) distribution and red lines (with diamonds and squares) to Log-normal (*γ*_1_ = 0 for solid lines and *γ*_1_ = 0.65 for dashed lines). For Log-normal distribution *σ* = 0.3. Note that for *γ*_1_ > 0 ED is constant (dashed red line with blue squares), i.e. ED = 0.15. (C) Dependence of ED on *γ*_2_ for all distributions of spine volumes. (D) ED as a function *θ*. Blue lines correspond to Gamma (n = 2) distribution and red lines (with diamonds and squares) to Log-normal (*γ*_2_ = 0.3 for solid lines and *γ*_2_ = 0.65 for dashed lines). For Log-normal distribution *σ* = 0.25. In panels (A) and (C) *θ* = 0.321 *μ*m^3^, and the following labels were used: Exponential distribution is shown as solid blue line, Gamma (n = 1) and Gamma (n = 2) are shown respectively as dashed green and dashdot cyan lines, Rayleigh distribution is represented by dotted black line, Log-logistic is shown as solid red line with circles, and Log-normal as solid black line with diamonds. The curves for Log-logistic and Log-normal correspond to different values of the parameters, respectively, *β* and *σ* that yield the minimal ED for a given *r* or *γ*_2_.

**Fig 6:**
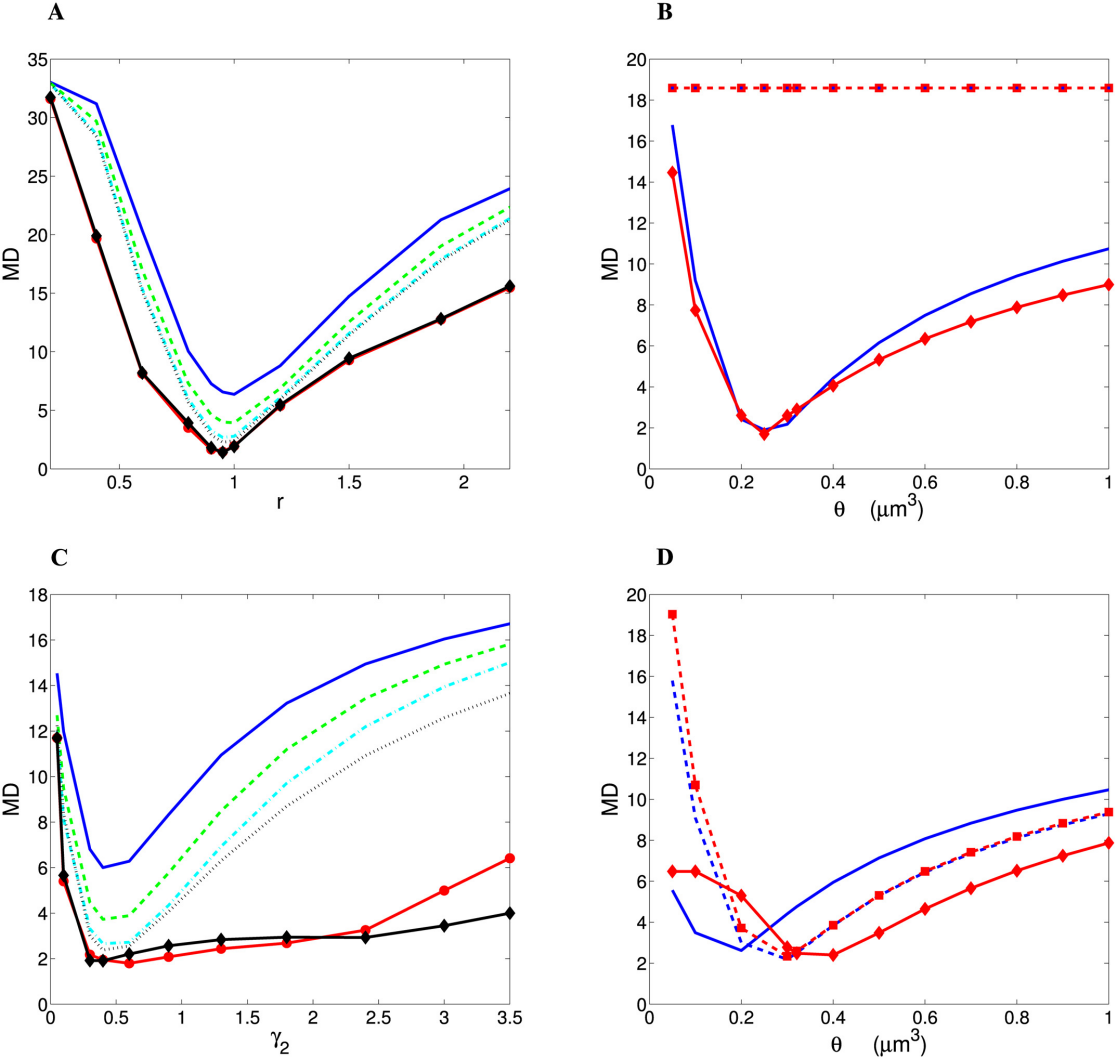
The same as in Fig 5 but for the more general Mahalanobis distance (MD) between optimal theoretical fractions and empirical fractions. (A) MD as a function of *r*, and (B) MD as a function of *θ* for wire minimization. (C) MD as a function of *γ*_2_, and (D) MD as a function of *θ* for spine economical maximization. In panel (D) *γ*_2_ = 0.2 for solid lines and *γ*_2_ = 0.5 for dashed lines.

The degree of similarity between the theory and the data is quantified by two different measures, their Euclidean distance ED, and their Mahalanobis distance MD (see Eqs 34 and 35). The latter is more general, as it takes variability in the data into account. Both measures yield qualitatively very similar results (compare Figs 5 and 6). For wire minimization principle, ED and MD depend biphasically (non-monotonically) on *r* if *γ*_1_ = 0 in a similar fashion for all distributions of spine volumes, with characteristic sharp minima for *r* ∼ 0.95, for which there are the best matches to the data (Figs 5A and 6A). The overall best results are achieved for Log-normal distribution (ED = 0.010, MD = 1.405), and other distributions especially those with short-tails produce higher ED and MD (Table 2). Outside the optimal value of *r* the values of ED and MD grow rather fast, and the agreement between theory and the experiment becomes weak (Figs 5A and 6A). If however, *γ*_1_ > 0, then ED and MD are both constant and relatively large (ED = 0.15, MD = 18.5), regardless of other parameters and distribution types (Figs 5B and 6B), which implies that similarity with the data is always very weak in this case.

On the other hand, for spine economical maximization ED and MD exhibit minima for *γ*_2_ < 1 (Figs 5C and 6C). Interestingly, neither the minimal values of ED and MD, nor associated with them the optimal values of *γ*_2_ vary much as we change the spine distribution type for a given threshold *θ* (Figs 5C and 6C). Specifically, the optimal *γ*_2_ is in the range 0.15–0.40 for *θ* = 0.100 *μ*m^3^, and 0.35–0.75 for *θ* = 0.321 *μ*m^3^ (Table 3). The minima of ED and MD for heavy-tailed distributions are much broader than for short-tailed, which suggests that heavy-tailed distributions can be more flexible in comparison to the data (Figs 5C and 6C). This follows from the fact that heavy-tailed distributions depend on an additional parameter that can be adjusted (Fig. A in Supporting S1 Text). Moreover, the best optimal cortical fractional volumes in Table 3 look very similar across different distributions of spine sizes. ED values for these best solutions are also very similar (Table 3). The smallest possible minimal value of ED is 0.038, and it is reached by four different distributions, independent of the asymptotic tail (Gamma n = 2, Rayleigh, Log-logistic, and Log-normal). (As a comparison, for a hypothetical situation when all theoretical optimal cortical components were uniformly distributed, i.e. each of them were 0.2, we would obtain ED = 0.342, which is about 10 times larger than the actual minimal ED in Table 3). The situation is different for the MD distance. Despite a high degree of similarity among the optimal fractional volumes across different distributions, MD discriminates these cases much better than ED (Table 3). Distributions having identical ED have in general different MD values (Table 3). The overall trend is such that the best fits to the empirical fractional volumes are given by the distributions of spine sizes with long-tails. The smallest possible minimal MD is achieved for Log-normal distribution (MD = 1.788; Table 3). Because of its higher generality, only the MD measure is used subsequently for comparing theoretical predictions with the data.

**Table 3:** The best optimal theoretical fractional volumes and related parameters in the cortex for “spine economical maximization” principle. The optimal fractions correspond to either the minimal Euclidean distance (ED) or Mahalanobis distance (MD) between theory and data (given in bold face).

Spine size distribution	*θ*	Optimal parameters								ED	MD
		*x*	*y*	*s*	*g*	*c*	$\bar{u}$	*P*	*γ*_2_		
Exponential	0.100	0.374	0.374	0.119	0.118	0.014	0.615	0.850	0.25	**0.043**	1.982
	0.100	0.374	0.374	0.119	0.118	0.014	0.615	0.850	0.25	0.043	**1.982**
	0.321	0.398	0.398	0.093	0.102	0.009	0.599	0.585	0.50	**0.050**	5.913
	0.321	0.397	0.397	0.098	0.097	0.010	0.678	0.623	0.45	0.051	**5.883**
Gamma (n = 1)	0.100	0.366	0.366	0.129	0.123	0.016	0.626	0.959	0.20	**0.051**	2.353
	0.100	0.366	0.366	0.129	0.123	0.016	0.626	0.959	0.20	0.051	**2.353**
	0.321	0.388	0.388	0.098	0.116	0.011	0.520	0.650	0.60	**0.039**	3.886
	0.321	0.385	0.385	0.111	0.107	0.012	0.660	0.746	0.45	0.042	**3.597**
Gamma (n = 2)	0.100	0.370	0.370	0.136	0.110	0.015	0.778	0.993	0.15	**0.056**	2.554
	0.100	0.370	0.370	0.136	0.110	0.015	0.778	0.993	0.15	0.056	**2.554**
	0.321	0.382	0.382	0.101	0.122	0.012	0.495	0.692	0.65	**0.038**	2.914
	0.321	0.380	0.380	0.112	0.116	0.013	0.589	0.774	0.50	0.040	**2.507**
Rayleigh	0.100	0.361	0.361	0.127	0.135	0.017	0.534	0.973	0.20	**0.056**	3.306
	0.100	0.371	0.371	0.136	0.108	0.015	0.806	0.988	0.15	0.056	**2.645**
	0.321	0.380	0.380	0.102	0.125	0.013	0.486	0.710	0.60	**0.038**	2.555
	0.321	0.378	0.378	0.113	0.118	0.013	0.585	0.789	0.45	0.040	**2.284**
Log-logistic	0.100	0.377	0.377	0.106	0.126	0.013	0.498	0.747	0.40	**0.039**	2.152
	0.100	0.377	0.377	0.106	0.126	0.013	0.498	0.747	0.40	0.039	**2.152**
	0.321	0.383	0.383	0.102	0.120	0.012	0.511	0.695	0.75	**0.038**	2.999
	0.321	0.372	0.372	0.114	0.127	0.015	0.524	0.824	0.60	0.043	**1.793**
Log-normal	0.100	0.381	0.381	0.098	0.128	0.013	0.445	0.675	0.30	**0.038**	2.794
	0.100	0.372	0.372	0.120	0.120	0.015	0.603	0.868	0.20	0.045	**1.880**
	0.321	0.383	0.383	0.101	0.121	0.012	0.499	0.688	0.55	**0.038**	3.005
	0.321	0.372	0.372	0.115	0.126	0.014	0.535	0.828	0.35	0.043	**1.788**

For log-logistic distribution the minimal ED and MD were obtained for the parameter *β* = 1.5 if *θ* = 0.100, and if *θ* = 0.321 then *β* = 3.0 for minimal ED and *β* = 4.0 for minimal MD. For log-normal distribution the minimal ED and MD were reached for *σ* = 0.75 if *θ* = 0.100, and if *θ* = 0.321 then *σ* = 0.25 for minimal ED and MD.

The degree of the sensitivity of the optimal cortical fractions on the threshold *θ* for spine formation is qualitatively mostly similar for both principles of cortical organization considered here (Fig 5B and 5D and Fig 6B and 6D), except for the case of wire minimization with *γ*_1_ > 0, for which ED, MD, and cortical fractions are independent of *θ* but are not realistic (Figs 5B and 6B). Indeed, for wire volume minimization (with *γ*_1_ = 0) and spine economical maximization, ED and MD depend very strongly on *θ* if *θ* < 0.2 *μ*m^3^ (Figs 5B and 5D and 6B and 6D). However, if *θ* > 0.2 *μ*m^3^ this dependence is milder in both cases (Figs 5B and 5D and 6B and 6D).

### Optimal spine sizes and probability of spine formation: Wire minimization vs. spine economical maximization

Optimization of the fitness function *F* also yields optimal average spine volume $\bar{u}$ and indirectly the conditional probability of spine formation *P* (see the Methods). We consider the results for pure wire minimization and pure spine economy maximization.

**Fig 7:**
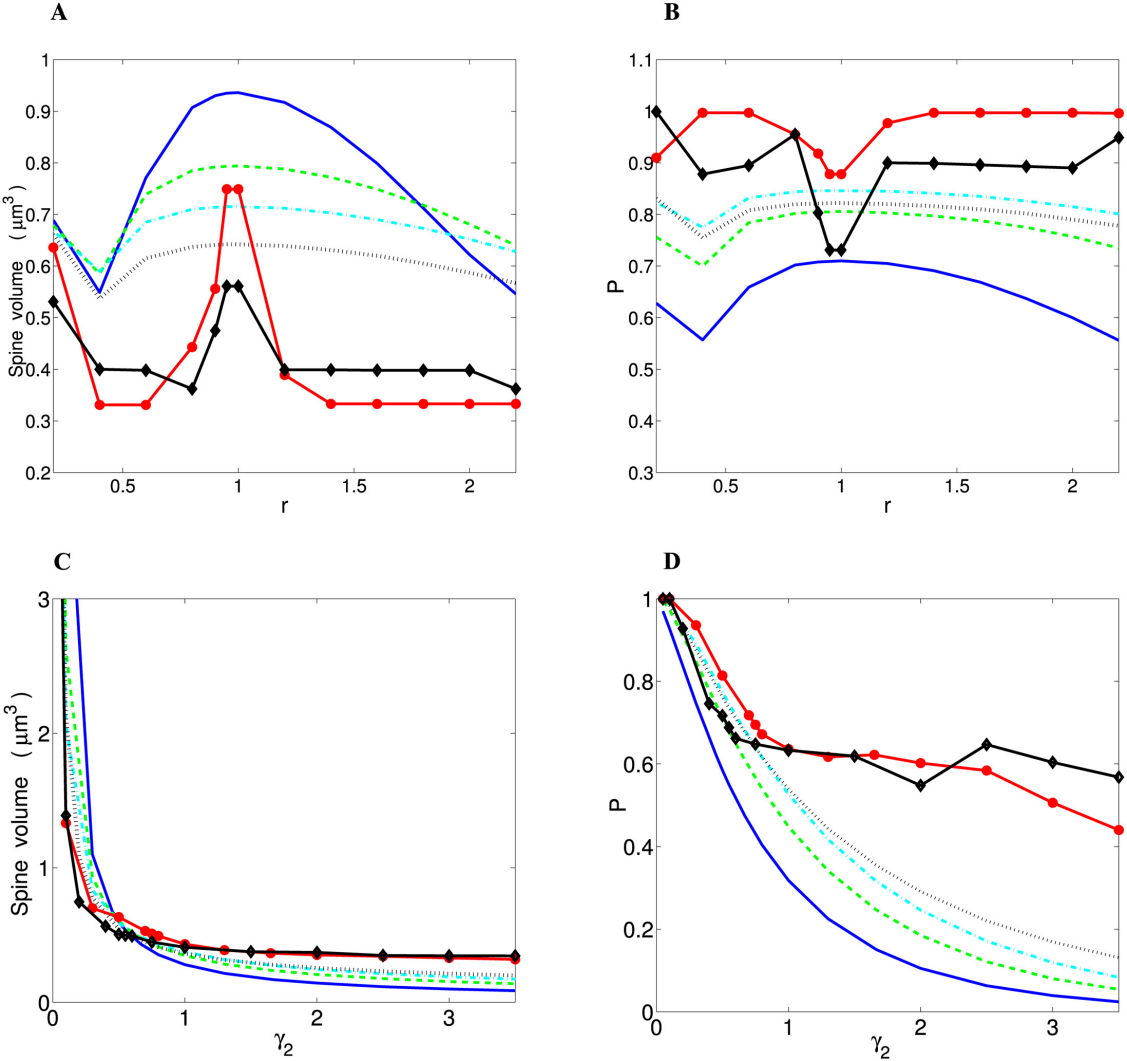
Optimal average spine volume $\bar{u}$ and conditional probability of spine formation *P* for different distributions of spine sizes. Panels (A) and (B) refer to “wire volume minimization”, whereas panels (C) and (D) correspond to “spine economical maximization”. (A) Non-monotonic dependence of spine volume $\bar{u}$ and (B) conditional probability *P* on *r* for wire fractional volume minimization (*γ*_1_ = 0). (C) Spine volume $\bar{u}$ and (D) conditional probability *P* decrease monotonically with increasing the exponent *γ*_2_. For all panels the same labels for curves corresponding to a given distribution were used, and they are identical to the labels used in Fig 5A and 5C. The curves for Log-logistic and Log-normal correspond to different values of the parameters, respectively, *β* and *σ* that yield the minimal ED for a given *r* or *γ*_2_. For all panels *θ* = 0.321 *μ*m^3^.

As was previously noted, for wire minimization principle (*f* = 1 in Eq 1) with *γ*_1_ > 0, we obtain $\bar{u}\;\mapsto \;\infty$, and consequently *P* = 1, both of which are unrealistic. For wire minimization with *γ*_1_ = 0 (wire volume minimization), both $\bar{u}$ and *P* depend non-monotonically on the parameter *r* (Fig 7A and 7B). For short-range distributions of spine size, $\bar{u}$ and *P* are positively correlated, whereas for the distributions with heavy-tails these two quantities are anti-correlated. Thus for wire minimization there is no clear one-to-one correspondence between average spine size and conditional probability of spine formation. Among all the distributions, for the optimal value of *r* (*r* ≈ 0.95), the heavy-tailed Log-normal produces the most realistic spine volume $\bar{u}=0.24-0.56\mu {\text{m}}^{3}$, i.e. the closest to the empirical values (0.2–0.4 *μ*m^3^ for human and macaque monkey [41, 42]), regardless of the value of threshold *θ* (Table 2; Fig 7A). For short-tail distributions the values of $\bar{u}$ are strongly threshold *θ*-dependent (Table 2).

In contrast, for spine economical maximization (*f* = 0 in Eq 1) the quantities $\bar{u}$ and *P* depend monotonically on the exponent *γ*_2_, and thus are positively correlated for all distributions, i.e. small spine volumes are generally associated with low probabilities *P* and vice versa (Fig 7C and 7D). For optimal values of *γ*_2_ (< 1) the average spine volumes have very similar values regardless of the spine size distribution and the threshold *θ* (Gamma n = 2 and Rayleigh distributions are exceptions), typically: $\bar{u}=0.44-0.67\mu {\text{m}}^{3}$ (Table 3), which is also close to the experimental data [41, 42].

### Optimal fractional volumes of cortical components: Combined “wire min and spine max” meta-principle

**Fig 8:**
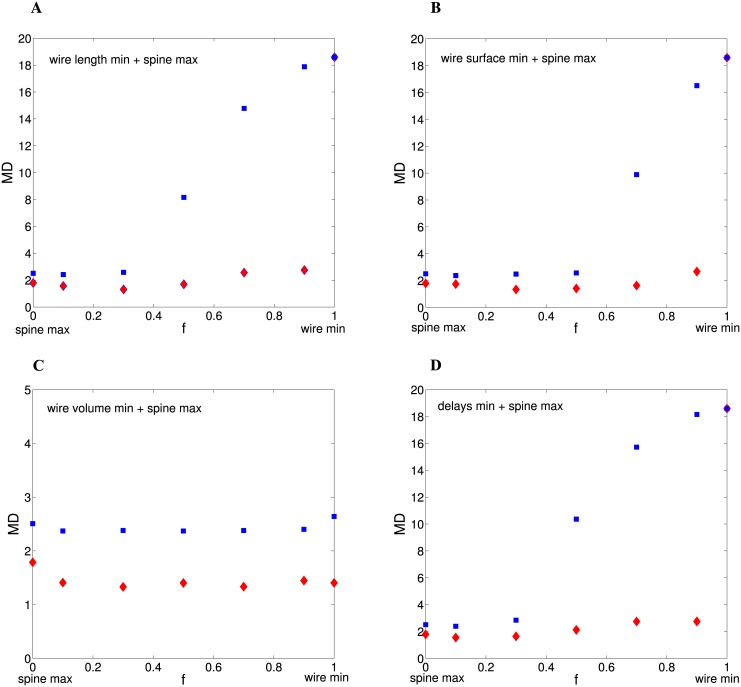
The minimal value of MD as a function the control parameter *f* for the combined “spine economy max and wire min” principle. Blue squares correspond to Gamma (n = 2) distribution of spine sizes and red diamonds to Log-normal distribution. The notion of spine economy max is mixed with different types of wire cost min principle: wire length in (A), wire surface area in (B), wire volume in (C), and conduction delays in (D). In almost all panels MD has extremely shallow minima for *f* ∼ 0.1–0.3, and then MD increases either weakly or abruptly as *f* increases further (for *f* = 1 MD is the same for both distributions of spine sizes). The exception is the mixture of wire volume min and spine max (panel C), where MD is practically constant. For all panels *θ* = 0.321 *μ*m^3^.

Now we consider the scenario when wire minimization and spine economical maximization notions are mixed together simultaneously, and optimal fractional volumes are obtained by minimization of the meta fitness function in Eq (1) with the control parameter 0 < *f* < 1. In general, the best results in terms of agreement with the empirical data are reached for the cases when spine economy rule dominates in the meta fitness function, i.e. *f* ≪ 1, (Fig 8; Tables 4–6). More precisely, MD distance has very shallow minima in the range *f* ∼ 0.1–0.3 (Fig 8). The worst results are found for the cases when wire minimization rule prevails (*f* ↦ 1), and intermediate results are obtained for the balanced case when spine economy max and wire min are treated with equal weights, i.e. *f* ∼ 0.5 (Fig 8 and Tables 4–6). However, there is an exception to this tendency, namely, the mixture of wire volume minimization and spine economy maximization, which yields invariant results and good similarity with the data regardless of the value of mixing ratio *f* (Fig 8C and Tables 4–6).

**Table 4:** The best optimal theoretical fractional volumes and related parameters in the cortex for the combined “wire min + spine max” principle with control parameter *f* = 0.1. The optimal fractions correspond to the minimal Mahalanobis distance (MD) between theory and data.

Principle type/spine distr.	Optimal parameters									MD
	*x*	*y*	*s*	*g*	*c*	$\bar{u}$	*P*	*r*	*γ*_2_	
**Wire length min + spine max** (*γ*_1_ = 2/3)										
Exponential	0.399	0.396	0.097	0.098	0.010	0.655	0.612	0.99	1.00	5.885
Gamma (n = 1)	0.395	0.377	0.107	0.110	0.012	0.612	0.717	0.90	0.95	3.558
Gamma (n = 2)	0.393	0.368	0.110	0.117	0.013	0.569	0.759	0.85	0.95	2.416
Rayleigh	0.395	0.362	0.110	0.120	0.013	0.553	0.768	0.80	0.90	2.117
Log-logistic	0.400	0.344	0.116	0.126	0.015	0.544	0.844	0.65	0.90	1.277
Log-normal	0.386	0.353	0.112	0.134	0.015	0.480	0.825	0.80	0.80	1.565
**Wire surface min + spine max** (*γ*_1_ = 1/3)										
Exponential	0.402	0.393	0.100	0.096	0.010	0.696	0.631	0.95	0.70	5.903
Gamma (n = 1)	0.394	0.377	0.108	0.109	0.012	0.629	0.728	0.90	0.70	3.549
Gamma (n = 2)	0.396	0.364	0.111	0.116	0.013	0.589	0.774	0.80	0.70	2.375
Rayleigh	0.399	0.358	0.111	0.118	0.013	0.576	0.784	0.75	0.65	2.105
Log-logistic	0.398	0.346	0.117	0.125	0.015	0.550	0.850	0.65	0.70	1.280
Log-normal	0.396	0.347	0.104	0.138	0.014	0.423	0.759	0.65	0.80	1.741
**Wire volume min + spine max** (*γ*_1_ = 0)										
Exponential	0.399	0.396	0.099	0.096	0.010	0.692	0.629	0.98	0.45	5.898
Gamma (n = 1)	0.398	0.374	0.107	0.110	0.012	0.617	0.721	0.85	0.50	3.553
Gamma (n = 2)	0.395	0.365	0.112	0.115	0.013	0.598	0.781	0.80	0.50	2.371
Rayleigh	0.397	0.359	0.113	0.117	0.013	0.590	0.792	0.75	0.45	2.111
Log-logistic	0.399	0.345	0.117	0.125	0.015	0.548	0.848	0.60	0.55	1.277
Log-normal	0.400	0.343	0.110	0.133	0.015	0.474	0.798	0.60	0.45	1.409
**Delays min + spine max** (*γ*_1_ = 5/6)										
Exponential	0.398	0.398	0.096	0.099	0.010	0.642	0.606	1.00	1.15	5.887
Gamma (n = 1)	0.390	0.382	0.106	0.111	0.012	0.597	0.708	0.95	1.10	3.588
Gamma (n = 2)	0.393	0.367	0.111	0.117	0.013	0.580	0.768	0.85	1.05	2.393
Rayleigh	0.392	0.366	0.108	0.121	0.013	0.537	0.755	0.85	1.05	2.164
Log-logistic	0.396	0.348	0.114	0.127	0.014	0.530	0.830	0.70	1.05	1.295
Log-normal	0.390	0.356	0.108	0.132	0.014	0.469	0.775	0.80	1.00	1.553

All the results correspond to *θ* = 0.321.

**Table 5:** The best optimal theoretical fractional volumes and related parameters in the cortex for the combined “wire min + spine max” principle with control parameter *f* = 0.5. The optimal fractions correspond to the minimal Mahalanobis distance (MD) between theory and data.

Principle type/spine distr.	Optimal parameters									MD
	*x*	*y*	*s*	*g*	*c*	$\bar{u}$	*P*	*r*	*γ*_2_	
**Wire length min + spine max** (*γ*_1_ = 2/3)										
Exponential	0.453	0.453	0.013	0.080	0.001	0.115	0.061	1.00	2.95	15.22
Gamma (n = 1)	0.421	0.433	0.036	0.107	0.004	0.212	0.195	1.05	2.75	11.75
Gamma (n = 2)	0.399	0.413	0.059	0.121	0.007	0.290	0.356	1.05	2.60	8.157
Rayleigh	0.393	0.407	0.065	0.127	0.008	0.299	0.405	1.05	2.55	7.047
Log-logistic	0.392	0.359	0.099	0.137	0.014	0.409	0.706	0.90	2.40	2.118
Log-normal	0.399	0.353	0.110	0.125	0.014	0.520	0.777	0.90	2.10	1.692
**Wire surface min + spine max** (*γ*_1_ = 1/3)										
Exponential	0.421	0.421	0.040	0.114	0.005	0.215	0.225	1.00	2.00	10.94
Gamma (n = 1)	0.395	0.395	0.079	0.122	0.010	0.389	0.509	1.00	1.85	5.508
Gamma (n = 2)	0.380	0.380	0.109	0.118	0.013	0.558	0.751	1.00	1.70	2.566
Rayleigh	0.392	0.368	0.102	0.125	0.013	0.486	0.710	0.95	1.65	2.436
Log-logistic	0.404	0.336	0.114	0.131	0.015	0.500	0.835	0.85	1.60	1.350
Log-normal	0.395	0.347	0.110	0.133	0.015	0.475	0.804	0.90	1.40	1.414
**Wire volume min + spine max** (*γ*_1_ = 0)										
Exponential	0.398	0.398	0.097	0.098	0.010	0.655	0.613	1.00	0.55	5.886
Gamma (n = 1)	0.403	0.369	0.107	0.109	0.012	0.617	0.721	0.95	0.60	3.566
Gamma (n = 2)	0.395	0.364	0.112	0.115	0.013	0.598	0.781	0.95	0.60	2.370
Rayleigh	0.394	0.363	0.111	0.119	0.013	0.567	0.777	0.95	0.55	2.107
Log-logistic	0.400	0.344	0.116	0.126	0.015	0.544	0.844	0.90	0.70	1.278
Log-normal	0.401	0.340	0.111	0.133	0.015	0.478	0.816	0.90	0.40	1.403
**Delays min + spine max** (*γ*_1_ = 5/6)										
Exponential	0.463	0.463	0.008	0.065	0.001	0.097	0.036	1.00	3.40	16.15
Gamma (n = 1)	0.438	0.438	0.026	0.096	0.002	0.183	0.135	1.00	3.10	13.34
Gamma (n = 2)	0.419	0.419	0.045	0.113	0.005	0.249	0.257	1.00	2.95	10.36
Rayleigh	0.410	0.410	0.053	0.121	0.006	0.265	0.317	1.00	2.90	8.918
Log-logistic	0.396	0.345	0.105	0.139	0.015	0.420	0.765	0.85	2.50	1.761
Log-normal	0.405	0.337	0.100	0.144	0.014	0.383	0.732	0.80	2.40	2.124

All the results correspond to *θ* = 0.321.

**Table 6:** The best optimal theoretical fractional volumes and related parameters in the cortex for the combined “wire min + spine max” principle with control parameter *f* = 0.9. The optimal fractions correspond to the minimal Mahalanobis distance (MD) between theory and data.

Principle type/spine distr.	Optimal parameters									MD
	*x*	*y*	*s*	*g*	*c*	$\bar{u}$	*P*	*r*	*γ*_2_	
**Wire length min + spine max** (*γ*_1_ = 2/3)										
Exponential	0.414	0.414	0.172	0.000	0.000	22077.8	1.000	1.00	0.10	18.45
Gamma (n = 1)	0.489	0.489	0.001	0.020	0.000	0.086	0.005	1.00	6.40	18.33
Gamma (n = 2)	0.484	0.484	0.003	0.029	0.000	0.118	0.012	1.00	6.20	17.88
Rayleigh	0.471	0.471	0.008	0.050	0.000	0.157	0.038	1.00	6.10	16.69
Log-logistic	0.414	0.414	0.172	0.000	0.000	22073.2	1.000	1.00	0.10	18.45
Log-normal	0.401	0.340	0.093	0.151	0.014	0.332	0.684	0.35	5.50	2.751
**Wire surface min + spine max** (*γ*_1_ = 1/3)										
Exponential	0.487	0.487	0.001	0.025	0.000	0.060	0.005	1.00	5.20	18.13
Gamma (n = 1)	0.478	0.478	0.004	0.040	0.000	0.107	0.018	1.00	4.90	17.38
Gamma (n = 2)	0.468	0.468	0.009	0.054	0.001	0.146	0.040	1.00	4.65	16.51
Rayleigh	0.454	0.454	0.017	0.074	0.001	0.181	0.084	1.00	4.60	15.07
Log-logistic	0.402	0.339	0.095	0.150	0.014	0.341	0.696	0.75	4.20	2.622
Log-normal	0.402	0.347	0.092	0.145	0.013	0.348	0.664	0.85	3.80	2.670
**Wire volume min + spine max** (*γ*_1_ = 0)										
Exponential	0.398	0.398	0.097	0.099	0.010	0.651	0.611	1.00	1.10	5.886
Gamma (n = 1)	0.386	0.386	0.108	0.109	0.012	0.628	0.728	1.00	1.20	3.591
Gamma (n = 2)	0.404	0.355	0.112	0.115	0.013	0.601	0.783	0.95	1.20	2.401
Rayleigh	0.403	0.354	0.111	0.119	0.013	0.568	0.779	0.95	1.00	2.126
Log-logistic	0.394	0.350	0.114	0.127	0.015	0.529	0.829	0.95	1.60	1.311
Log-normal	0.396	0.349	0.109	0.132	0.014	0.472	0.788	0.95	0.70	1.447
**Delays min + spine max** (*γ*_1_ = 5/6)										
Exponential	0.425	0.403	0.171	0.001	0.000	2095.2	1.000	0.50	0.10	18.37
Gamma (n = 1)	0.425	0.403	0.171	0.001	0.000	2091.7	1.000	0.50	0.10	18.37
Gamma (n = 2)	0.487	0.487	0.002	0.024	0.000	0.111	0.008	1.00	6.70	18.15
Rayleigh	0.475	0.475	0.006	0.043	0.000	0.151	0.029	1.00	6.70	17.11
Log-logistic	0.425	0.403	0.171	0.001	0.000	2091.7	1.000	0.50	0.10	18.37
Log-normal	0.401	0.340	0.093	0.151	0.014	0.332	0.684	0.40	5.60	2.751

All the results correspond to *θ* = 0.321.

For the case when spine economy predominates (*f* = 0.1) in *F*, we obtain that mixing spine max with a small fraction of any type of wire min rule (arbitrary *γ*_1_) gives essentially the same results for MD (Table 4), and they are very similar to the results for pure wire volume minimization (Table 2) and pure spine economy maximization (Table 3). This means that for this particular scenario all kinds of wire minimization rule, such as length min, surface area min, volume min, or delays min, are equally reasonable, but they are less important than spine economy. This, however, is not the case when wire minimization prevails in the mixing of notions, or when there is a balance between them (cases *f* = 0.9 and *f* = 0.5, respectively). In these scenarios, wire volume min (and occasionally wire surface min) gives noticeably better results than either wire length min or delays min (Tables 5 and 6). However, it has to be stressed that mixing together spine economy rule with any of the wire cost rules with *γ*_1_ > 0 (wire length, wire surface, delays), at any ratio, always gives better results than for these wire cost rules alone, i.e., for *f* = 1 (Fig 8A, 8B and 8D).

Another interesting feature of the results in Tables 4–6 is that the best agreements with the data are almost always recorded for the distributions of spine sizes with long tails. This trend is conserved for a large spectrum of the values of *f*, however, as *f* ↦ 1 (the notion of wire min dominates in *F*) the similarity to the data becomes weaker or even non-existent (see Fig 8 for Log-normal distribution).

To summarize, mixing the rules of spine economy max with wire min does not give significantly better results than for pure spine economy max or pure wire volume min. At best, such a mixing yields marginally better results, but only if the spine component prevails (*f* ≪ 1) in the meta fitness function *F*.

## Discussion

### Hierarchy in cortical composition and the rule “powers of 1/3”

Experimental data analyzed in this paper indicate that the distribution of the basic components in the gray matter of cerebral cortex is relatively stable across mammalian species (Table 1; Fig 2). Axons and dendrites occupy on average similar fractions ∼ 1/3 of cortex volume, spines and glia/astrocytes take each about 1/10 of the cortex, and capillaries constitute roughly ∼ 1/100 of the cortical space. These numbers in themselves are interesting, because they form a special hierarchy of dependencies, being approximately integer powers of 1/3 (Fig 1). Specifically, the content of spines or glia/astrocytes is roughly equal to the square of axon (or dendrite) content, and capillary content in turn is approximately equal to the square of spine content (Table 1). This component hierarchy may be somehow important for efficient cortical computation, and thus worth investigating.

### Summary of the main theoretical results: The importance of spine economy principle

Hierarchy in cortical composition was a motivation for theoretical considerations about principles governing organization of the cerebral cortex. The goal was to provide a theoretical explanation of this hierarchy starting from a neurobiologically plausible yet simple principle. Two basic principles are considered: different forms of a standard neural wire minimization [3, 7–9, 43], and the new one proposed here called spine economical maximization. The latter rule is related to maximization of spine content in the cortex with simultaneous minimization of average spine size (which is supposed to reduce the metabolic cost). We also study a mixture of the two principles as a meta principle. The optimal outcomes of these models are compared to the experimental data, using two similarity measures (Euclidean and Mahalanobis distances).

This study shows that from many implementations of wire minimization concept, only the minimization of wire fractional volume (*γ*_1_ = 0) can give reasonable results that are close to the experimental data, if a free parameter (*r* in Eq 21) is chosen appropriately (compare Tables 1 and 2; Figs 5A and 6A). The other possibilities related to wire minimization (with *γ*_1_ > 0), such as minimization of wire length, its area, or conduction delays, yield unrealistic results: zeros for volume fractions of glia/astrocytes and capillaries (Fig 3A and 3C), resulting in relatively high values of ED and MD (Figs 5B and 6B), and infinite values of the average spine volume, which is clearly wrong. The last result follows from the fact that the minimal value of *F*_*w*_ in Eq (21) is precisely zero, which takes place only for $\bar{u}=\infty$ (other possibility with vanishing of axonal *x* and dendritic *y* fractions is forbidden, since that would imply that all fractions are zero, which would violate the fractions normalization constraint represented by Eq (31)).

On the other hand, the principle of spine economic maximization produces the fractional cortical volumes that are also close to the data (compare Tables 1 and 3), but they do not require such a careful tuning of a free parameter (*γ*_2_ in Eq 24), especially for the distributions of spine volume with heavy-tails (compare the scales in Fig 5C vs. 5A and in Fig 6C vs. 6A). Thus, both principles provide quantitatively similar results, however, maximization of the simple benefit-cost function *F*_*s*_ with spine content in the centerpiece (see Eq 24) produces a more robust outcome. This conclusion is consistent with suggestions that neural systems are not exclusively optimized for minimal wiring length or component placement [15, 16], and other factors or their combinations can be also involved [11–16]. This suggests that economic spine content maximization can possibly provide an additional and/or alternative mechanism that is used by evolution to regulate the efficiency of cortical circuits.

As a third possibility we consider the meta principle that combines spine economy with wiring cost, with some mixing ratio *f*. This mixing scenario improves greatly the results associated with minimization of wire length, wire surface area, and temporal delays (*γ*_1_ > 0), by making MD distances much smaller, but only when the spine economy dominates in the mixing of notions in the fitness function (Eq 1). Thus, clearly the presence of the principle of spine economical maximization is necessary to make the concepts of wire length and temporal delays minimizations to be relevant candidates for the explanation of cortical composition hierarchy (Table 4). However, it must be emphasized that mixing of wire volume min (*γ*_1_ = 0) with spine economy max does not produce noticeably better results (only a tiny improvement) in comparison to the cases when these two principles act in isolation (Fig 8C).

Finally, for all three scenarios (either *f* = 0, *f* = 1, or *f* between 0 and 1 in Eq 1), the best theoretical fractional volumes are generally obtained for distributions of spine volume with heavy-tails (Tables 2–6). In addition, many of these best solutions give optimal average spine volumes $\bar{u}$, which despite some variability, are in good agreement with experimental values, which are 0.35 *μ*m^3^ for human [41], and 0.3–0.4 *μ*m^3^ for macaque monkey [42]. Spine size correlates with synaptic weight [19], and hence variability in spine size that is observed among the best solutions is a positive feature, because it implies variability in synaptic weights, which in turn is necessary for brain function.

The models for pure wire volume minimization and for pure spine economy differ strongly in their predictions regarding the relationship between average spine size and conditional probability of spine formation *P* (Fig 7A and 7B vs. Fig 7C and 7D). While for wire volume minimization there is no clear one-to-one correspondence, for spine economy we obtain that small spines are associated with small *P* (Fig 7C and 7D). This means that for the latter principle very small spines are unlikely to form and thus are highly stochastic, even when an axon and dendrite are very close to each other. In contrast, large spines with sufficiently large energy capacity have high probability of forming stable synapses. Mathematically, this effect is achieved in the model by introducing the threshold *θ* for spine volume (see Eq 3 in the Methods). This theoretical result for spine economical maximization is in line with experimental observations, indicating high stochastic motility of small spines and structural stability of larger ones [19, 44–47].

### Previous directly related work

In the past there was only one directly related work associated with cortical composition and its theoretical basis [8]. In that study, the authors analyze only the combined optimal fractional volume of axons and dendrites (called wire), which turns out to be close to the empirical value. Chklovskii et al [8] used specific “thought experiments” to demonstrate that the optimal wire fraction can be derived from several equivalent principles, such as minimization of conduction delays, minimization of wiring length, and maximization of synaptic density. The present study is similar in spirit, i.e. in the expectation that fractional volumes are the result of some evolutionary optimization, but differs in the scope and details of what is actually optimized. Apart from considering different forms of wire minimization, we also investigate a new principle of spine economy, and its combination with wire cost (meta principle represented by Eq 1), and all three are analyzed in much more detail than in [8]. In particular, in this work we study five cortical components both neuronal and non-neuronal, in contrast to [8], who considered only a simple division wire vs. non-wire. Moreover, we provide explicit formulae for fractional volumes of different components and their mutual couplings, based on a concept of geometric probability, efficient transport for astrocytes, and some neuroanatomical observations.

### Other formulations of the problem related to spine economy

One may wonder why in the fitness function related to spine economy (Eq 24), the spine proportion (*s*) is maximized instead of (maybe more natural) numerical spine density (*ρ*_*s*_)? In fact, both possibilities are included in the fitness function, since these two quantities are linearly related ($s=\rho_{s}\bar{u}$). Thus the fitness function (Eq 24) can be equivalently written as $F_{s}∼\rho_{s}/{\bar{u}}^{\gamma_{2}-1}$, with the renormalized power of $\bar{u}$. However, since the best results are obtained for *γ*_2_ < 1, this new formulation implies that spine density and average spine volume both would have to be maximized, i.e., there would be no penalty on spine size. Thus, the original fitness function with spine proportion *s* seems to better capture the energy constraint, which suggests that *s* is a more primary variable than *ρ*_*s*_.

### Organization of local vs global circuits

This study considers local cortical circuits, presumably corresponding to a cortical column in size, and does not address the large-scale organization of the mammalian brain. In a present mathematical formulation of local circuits the notion of spatial scale is not taken explicitly into account. This is justified by the fact that locality means that all elements are sufficiently close to each other so that there should not be large detrimental delays in their communication. That temporal delays are not critical in local networks follows from a fact that diameters of intracortical axons and dendrites, directly related to velocities of propagated signals along them [48], are invariant with respect to brain size [14]. The situation is different for global long-range connections via white matter, which show a slight increase in average axon diameter with brain size [49]. Moreover these axons are myelinated, which enhances several-fold the speed of signal propagation in comparison to unmyelinated intracortical axons. These empirical facts indicate that for global brain organization the distance and delays are important constraints, and they were repeatedly used by many researchers to model large-scale organization of brain connectivity [7, 10, 15–17, 50]. Some of these studies initially showed that certain parts of the nervous system in macaque monkey (prefrontal areas) and in the nematode *C. elegans* are optimized for wiring length [7, 10, 50]. However, more recent studies demonstrated that wire length was not fully minimized across macaque and cat visual areas [17], and more importantly, global connections in the whole networks of macaque and *C. elegans* brains are far from being optimal for wire cost [15, 16]. The latter studies indicate that there might be other constraints on global brain organization, such as the requirement of short processing paths, which was proposed in the past [51], or some other combinations [3, 11–14].

The important issue is how to relate local organization in the cortex to global cortico-cortical connectivity. This is a challenging task, not only conceptually, but also from a methodological point of view. The approach presented here for the description of local networks relies on analytical optimization of Lagrange functions, and it differs considerably from the approaches used for studying patterns of long-range connections, which use mainly numerical algorithms of graph theory [13, 15, 16]. It is not clear how to combine the two approaches within a single mathematical framework. However, despite these technical difficulties, there is at least one quantity that can relate local and global organizations. This quantity is associated with the fraction of axons in the cortex *x*, which is composed of two contributions: local intracortical axons and endings of the long-range axons (via white matter). Empirical data indicate that the latter component is substantial [52], and it seems to be possible to find a mathematical formula relating the two contributions. Since the long-range part of *x* should be somehow correlated with the volume of axons in white matter, it may be feasible to make a connection between variables operating locally with those operating globally. In particular, one might try to derive from “first principles” the scaling relation between volumes of gray and white matters.

### Generalization of the optimality models

The principle of spine economy maximization (as well as other principles) was defined locally in this paper. This means that spatial correlations between different cortical components were neglected. One natural extension of this work is to include spatial dependence in the fitness functions (Eqs 1, 21 and 24), by considering more local circuits with slightly different properties that are coupled together. Such an approach would allow us, in principle, to model spatial plasticity effects and competition for space in the cortex. For example, it is known that learning modifies the structure of spines [19], and there are some indications that it also alters dendritic and axonal processes [53]. The interesting question here is how these two types of modifications are related to one another, and how they influence neighboring circuits.

Another possible extension is to include explicitly the temporal aspect in the equations. The current approach, without time, describes a mature “average” brain. Time dependence of cortical composition would allow us to model the effects associated with brain development. In particular, there are data on synaptic density development in different parts of the cortex across species (references in [27]). Many of these dependencies show that synaptic density acquires a maximum at some early developmental stage, and then it decays to adult (stable) values. It would be interesting to see if spine economy rule combined with time can generate such non-monotonic dependencies. The temporal aspect in the equations could formally be included in analogy with a Hamiltonian approach known from classical mechanics [54], i.e. the fitness function *F*_*s*_ could serve as a Hamiltonian of the local circuit.

### Conclusions

This study shows that hierarchical composition of local cortical circuits can be best explained by two different design principles. One is associated with a new proposition called here spine economical maximization, and another with neural wire volume minimization, and both give similar the best optimal solutions. In contrast, other principles related to wire minimization such as: wire length, wire surface area, or conduction delays minimizations do not yield reasonable results. Only when combined with spine economy rule, these other notions can equally well be fitted to the data under some conditions (spine economy contribution must dominate in the mixing of concepts in the meta-fitness function and/or spine size distribution must have a long tail). These results imply that for the efficiency of local circuits (i) wire volume may be more basic variable than wire length or temporal delays, (ii) spine economy principle may be an important concept, and (iii) we should pay more attention to spines, especially in a broader context of brain evolutionary design.

## METHODS

### Data gathering and analysis for cortical composition

#### The ethics statement does not apply to this study

Experimental data in Table 1 for cortical composition come from different sources. They are either directly taken from a source or calculated based on other related neuroanatomical data.

**Mouse data.** Data from [38], except for capillaries—data from [29].

**Rat data.** Data from [55], except for capillaries—data from [56].

**Rabbit data.** Fractional volumes are arithmetic means of the values for cortical spaces between and within dendrite bundles in the visual cortex of layers 2 and 3 [57].

**Cat data.** Density of axon length and dendrite length near the layer 3/4 of visual cortex was estimated as respectively 3.93 ± 0.8 *μ*m/*μ*m^3^ and 0.39 ± 0.08 *μ*m/*μ*m^3^ [52]. The fractional volumes of axons and dendrites were obtained by assuming axon and dendrite diameters as respectively 0.3 *μ*m [38] and 1.0 *μ*m [58]. Astrocyte data come from [59], and capillary data from [60].

**Macaque monkey data.** The fractional volume of dendrites was estimated in prefrontal cortex as 0.33 ± 0.19, based on the formula: (*π*/4)*ρ*_*n*_ *l*_*d*_ *d* ^2^, where the neuron density *ρ*_*n*_ = (1.0 ± 0.2)10^5^ mm^−3^ [61], average total dendrite length per neuron *l*_*d*_ = 3478 ± 99 *μ*m [62], and average dendrite diameter *d* = 1.1 ± 0.2 *μ*m [63, 64]. The volume fraction of spines was estimated as a product of average spine head volume 0.15±0.01 *μ*m^3^ (probably an underestimate for a whole spine volume) and spine density 0.30 ± 0.03 *μ*m^−3^ in prefrontal cortex [65]. Capillary fraction data come from visual cortex [30].

**Human data** Average spine volume (cingulate cortex) is 0.35±0.02 *μ*m^3^ [41] and density of asymmetric synapses (temporal cortex), presumably spines, is (4.23 ± 2.59)10^−1^ *μ*m^−3^ [66]. Average fraction of spine volume was estimated as the product of these two parameters. The ratio of cortical volumes taken by dendrites and by spines is 2.39, which comes from dividing total volumes of basal and apical dendrites (424.5 *μ*m^3^) and spines (177.3 *μ*m^3^) per neuron in cingulate cortex of 40 years old human [41]. The volume fraction of dendrites was estimated as a product of 2.39 and the volume fraction of spines. Astrocyte and capillary fractions data come from parietal cortex [67].

### Theoretical modeling

To find the optimal structural layout of the mature cerebral cortex we slightly simplify the analysis and consider its five major components that seem to be functionally important: axons, dendrites, spines, glia/astrocytes, and capillaries. In the considerations below we rely on a concept of geometric probability, which relates fractional volumes of cortical components with average probabilities of their encountering. In this mean-field approach, the details of neuronal or glial arborizations are not important.

#### Model of spine fractional volume

In a mature brain axons and dendrites are much more structurally stable than synapses (spines), which can change volume or even disappear relatively fast [44–47]. Consequently, it is assumed that axonal and dendritic fractions form two primary independent variables that set to a large degree the cortical layout. A third, independent variable is an average spine volume. Spine size is indirectly related to the amount of metabolic energy it uses, through Na/K-ATPase pumps located on spine membrane [21]. Bigger spines with larger surface area require more energy for pumping out Na^+^ ions and maintaining their concentration gradient than smaller spines. There is some evidence that dendritic spines are the major energy users in the cortex [25–27], and thus, their energy or alternatively their volume, seems to be an important variable. In this study, we focus explicitly on spine volume as an independent variable instead of spine energy, since there exist data on the distribution of spine sizes [41, 68, 69] or spine EPSP [70], and no data on spine energy distribution.

A synaptic connection between excitatory neurons, which are majority in the cortex [38], can potentially be generated in that cortical region where axonal and dendritic trees spatially overlap [71]. However, a physical vicinity of axons and dendrites may not be sufficient for the appearance of a spine in this location [72], which suggests an additional factor of possibly stochastic nature involved [69]. It is assumed here that this factor is associated with energy. Specifically, we require that a metabolic energy allocated to a spine must be above a certain threshold to form a stable spine (or equivalently that potential spine volume must be large enough). This requirement relates to the empirical fact that physiological processes need a minimal amount of energy to be activated [73], which seems to apply to dendritic spines, since they disappear during prolonged severe ischemia [74] and during excessive cooling of the tissue [75]. Thus, the average probability of finding a spine at some location is equal to the product of two average probabilities: that axons and dendrites are present there, and that a potential spine is larger than a certain threshold. On the other hand, based on the concept of geometric probability, the probability of finding a spine in the cortex is approximately equal to the fractional cortical volume occupied by spines, which is denoted as *s*. Mathematically, this means:

$$\begin{array}{c} s=Pxy, \end{array}$$ (2)

where the parameters *x* and *y* denote fractional volumes of axons and dendrites in the cortex or probabilities of their occurrence (i.e. 0 < *x*, *y* < 1). The product *xy* is the average probability that both axon and dendrite are present in a small cortical space. The symbol *P* is the conditional probability that a spine has volume *u* that is greater than the threshold *θ*. Formally, this probability is defined as

$$\begin{array}{c} P=\int_{\theta }^{\infty }H\left(u\right)du, \end{array}$$ (3)

where *H*(*u*) is the distribution (density of probability) of spine volumes *u*. One can view *P* as a conditional probability of spine formation. We consider five different types of volume distribution functions *H*(*u*), for which we obtain different forms of the probability $P(\bar{u})$ as a function of average spine volume $\bar{u}$ (see below).

#### Distributions of spine sizes

Empirical data show that spines can have widely different sizes, from very small ∼ 0.01 *μ*m^3^ to quite large ∼ 1.3 *μ*m^3^ [41, 76]. The distribution of their sizes has been fitted by two distinct functions with different asymptotic properties, either by gamma function with short-tail [41], or by log-normal with heavy-tail [69]. This suggests that there could also be other distributions, which are statistically indistinguishable from the above, that would fit the data equally well. For this reason and for a larger generality we consider five different distributions *H*(*u*) of spine volumes: three with short-tail and two with heavy-tail. For each distribution we provide explicit forms of the conditional probability *P* in terms of the average spine volume $\bar{u}$, which is defined as

$$\begin{array}{c} \bar{u}=\int_{0}^{\infty }H\left(u\right)udu. \end{array}$$ (4)

Exponential distribution.

This type of spine volume distribution has the form:

$$\begin{array}{c} H\left(u\right)=\alpha e^{-\alpha u} \end{array}$$ (5)

for *u* ≥ 0, where *α* is some positive constant. The average spine volume is $\bar{u}=1/\alpha$. The conditional probability *P* (defined in Eq 3) that a spine is greater than the threshold *θ* is

$$\begin{array}{c} P\left(\bar{u}\right)=e^{-\theta /\bar{u}}, \end{array}$$ (6)

i.e. it can be expressed as a function of $\bar{u}$. The latter feature applies to all size distributions considered in this paper (see below). For $\bar{u}/\theta \ll 1$ the probability $P(\bar{u})\ll 1$, whereas for $\bar{u}/\theta \gg 1$ we have $P(\bar{u})\approx 1$.

Gamma distribution.

The distribution of spine volume *H* is:

$$\begin{array}{c} H\left(u\right)=\frac{\alpha^{n+1}}{n!}u^{n}e^{-\alpha u} \end{array}$$ (7)

for *u* ≥ 0, where *α* is some positive constant. The average spine volume is $\bar{u}=\left(n+1\right)/\alpha$. The probability $P=\Gamma (n+1,\frac{(n+1)\theta }{\bar{u}})$, where Γ is the standard Gamma function. In the paper we consider two special cases, with *n* = 1 and *n* = 2, for which the probability *P* takes the following forms:

$$\begin{array}{c} P\left(\bar{u}\right)=(1+2\frac{\theta }{\bar{u}})\;e^{-2\theta /\bar{u}} \end{array}$$ (8)

for *n* = 1, and

$$\begin{array}{c} P\left(\bar{u}\right)=[1+3\frac{\theta }{\bar{u}}+\frac{9}{2}{\left(\frac{\theta }{\bar{u}}\right)}^{2}]\;e^{-3\theta /\bar{u}} \end{array}$$ (9)

for *n* = 2.

Rayleigh distribution.

The distribution of spine volume is given by

$$\begin{array}{c} H\left(u\right)=\frac{u}{\sigma^{2}}e^{-u^{2}/\left(2\sigma^{2}\right)} \end{array}$$ (10)

for *u* ≥ 0. The average spine volume is $\bar{u}=\sqrt{\frac{\pi }{2}}\sigma$. The probability *P* that spine is larger than the threshold *θ* is

$$\begin{array}{c} P\left(\bar{u}\right)=e^{-\frac{\pi }{4}{\left(\theta /\bar{u}\right)}^{2}}. \end{array}$$ (11)

Log-logistic distribution.

This type of distribution has a heavy tail and is represented by

$$\begin{array}{c} H\left(u\right)=\frac{\beta }{\alpha }\frac{{\left(u/\alpha \right)}^{\beta -1}}{{\left[1+{\left(u/\alpha \right)}^{\beta }\right]}^{2}} \end{array}$$ (12)

for *u* ≥ 0, where *α* is a positive constant and *β* > 1. Note that *H*(*u*) decays as a power law for asymptotically large *u*. The average spine volume is $\bar{u}=\alpha \pi /\left(\beta \sin\left(\pi /\beta \right)\right)$. The probability *P* in terms of $\bar{u}$ is given by

$$\begin{array}{c} P\left(\bar{u}\right)=\frac{{\bar{u}}^{\beta }}{{\bar{u}}^{\beta }+{\tilde{\theta }}^{\beta }}, \end{array}$$ (13)

where $\tilde{\theta }$ is the renormalized threshold, i.e. $\tilde{\theta }=\theta \frac{\left(\pi /\beta \right)}{\sin(\pi /\beta )}$. Eq (13) is known as Hill equation and is often used in biochemistry when there are cooperative phenomena between different molecules. Note that for sufficiently large exponent *β*, the probability *P* ≪ 1 if $\bar{u}<\tilde{\theta }$, and *P* ∼ 1 if $\bar{u}>\tilde{\theta }$.

Log-normal distribution.

The distribution of spine volume in this case also has a heavy tail, and it is given by

$$\begin{array}{c} H\left(u\right)=\frac{1}{\sqrt{2\pi }\sigma u}\exp[-\frac{{\left(\ln u-\mu \right)}^{2}}{2\sigma^{2}}], \end{array}$$ (14)

where *μ* and *σ* are some parameters (*σ* > 0). The average spine volume $\bar{u}$ is $\bar{u}=\exp\left(\mu +\sigma^{2}/2\right)$. The probability that a spine has larger volume than the threshold *θ* is

$$\begin{array}{c} P\left(\bar{u}\right)=\frac{1}{2}\;[1-\text{erf}\;(\frac{\ln\left(\theta /\bar{u}\right)+\sigma^{2}/2}{\sqrt{2}\sigma })], \end{array}$$ (15)

where erf(…) is the standard error function. Note that for *σ* ≪ 1, we have *P* ∼ 1 if $\bar{u}/\theta \gg 1$ and *P* ≪ 1 if $\bar{u}/\theta \ll 1$.

#### Model of glia and capillary fractional volumes

The model of glia and capillary fractions presented below relies on empirical evidence that cortical neurons with their synapses are spatially coupled to glia (astrocytes) and microvasculature. Specifically, it was shown that neuron density for mouse cortex and macaque monkey visual cortex correlate with vascular length density [29, 30]. The changes in astrocyte density are to some extent related to changes in capillary density across layers of mouse somatosensory cortex [31] (see below). Moreover, developmental data for cat visual cortex show a strong spatio-temporal coupling between capillary length density and synaptic density [77].

Extended branching processes of astrocytes resemble dendritic trees of neurons [78]. The endings of these processes physically connect with spines by wrapping around their surface to provide spines with metabolic substrates and glutamate from capillaries, and to remove waste products [78]. The transport of metabolites to and from spines along astrocytes should be effective, i.e. sufficiently fast and the least energy consuming, otherwise spines and thus local neural circuits would not get enough energy on time and as a result would underperform their functions. (Although glia use much less energy than neurons [21], they nevertheless should minimize their metabolic needs for the overall brain efficiency). This suggests that the total length of astrocyte processes should be as small as possible. Consequently, we can treat an astrocyte as a minimal spanning tree along its target points, i.e., spines. It can be shown mathematically that in a minimal tree formalism, the total length of the tree connecting *n* target points or branch points scales asymptotically as *n* ^2/3^ [79, 80]. The important point is that this result applies universally to any transportation network that works efficiently. Additionally, because of the physical units consistency, the total length of the tree should scale with enclosing volume *V* as *V* ^1/3^. Thus, the total length *L* of processes of a single astrocyte connecting *N*_*s*_ spines should be minimized when

$$\begin{array}{c} L=bN_{s}^{2/3}V^{1/3}, \end{array}$$ (16)

where *b* is some constant. It can be shown theoretically under general conditions that *b* = (3/(4*π*))^1/3^ [5]. This value was also found empirically for neural dendritic trees [5], and because of the structural similarity between branching patterns of astrocytes and dendrites, this particular value of *b* is also adopted here. The parameter *V* in Eq (16) is the cortical volume enclosing a single astrocyte and *N*_*s*_ spines. This “domain volume” is defined by three-dimensional boundaries of an astrocyte, and it is much bigger than the actual volume of an astrocyte because it includes also other cortical components (spines, dendrites, axons, etc) contained within these boundaries. The critical feature of astrocyte domains is that neighboring astrocytes essentially do not overlap, which means that each domain contains only one astrocyte cell that influences synaptic spines only from that particular domain. This property of astrocytes spatial arrangement is called domain organization ([78, 81]).

The main contribution to astrocyte volume comes from astrocyte free processes (astrocyte soma and astrocyte perivascular sheath constitute only 28% and 7% of the total astrocyte volume; [67]). Assuming a cylindrical geometry for these extended processes, the total volume of an astrocyte *V*_*as*_ can be approximated as $V_{as}=\left(\pi /4\right)Ld_{as}^{2}$, where *d*_*as*_ is the average diameter of all free processes. The value of *d*_*as*_ can be estimated based on data for volume (*V*_*pr*_ = 350 *μ*m^3^) and surface area (*S*_*pr*_ = 1650 *μ*m^2^) of astrocyte processes (without soma) in the cat sensorimotor cortex [59]. Using a familiar formula *d*_*as*_ = 4*V*_*pr*_/*S*_*pr*_, we obtain *d*_*as*_ = 0.85 *μ*m. One can expect that the value of *d*_*as*_ only very weakly depends on brain size, as it is the case for diameters of the thickest processes, which are 2.2 *μ*m for mouse, and 2.9 *μ*m for human [82], despite four orders of magnitude difference in brain volumes of these mammals (e.g. [22]). For that reason, the value *d*_*as*_ = 0.85 *μ*m is kept constant for all computations performed in this study, and it is the only parameter that is fixed in the model.

Because of the astrocyte domains segregation, the volume fraction of astrocytes can be defined as *g* = *V*_*as*_/*V*, which combined with Eq (16) yields

$$\begin{array}{c} g=a\rho_{s}^{2/3}=\frac{as^{2/3}}{{\bar{u}}^{2/3}}, \end{array}$$ (17)

where $a=\left(\pi /4\right)bd_{as}^{2}=0.352\mu {\text{m}}^{2}$, spine density *ρ*_*s*_ = *N*_*s*_/*V*, and in the last equality we used the fact that density *ρ*_*s*_, spine proportion *s*, and average spine volume $\bar{u}$ are related by

$$\begin{array}{c} s=\rho_{s}\bar{u}. \end{array}$$ (18)

The spatial separation between capillaries and spines, and between capillaries and astrocytes is relatively small, which presumably ensures a high efficiency of energy delivery. For example, a typical distance between capillaries and spines in mouse cortex is roughly 13 *μ*m [74]. Many astrocyte processes are either in the vicinity or directly touch capillaries [78]. Even astrocyte somata is close to microvasculature, with mean spacing between them 6–10 *μ*m in mouse somatosensory cortex, which at some locations can be down to ∼ 1 *μ*m [31]. For a comparison, an intercapillary distance is generally much larger: 32–43 *μ*m for mouse cortex [83], and 58 *μ*m for human cortex [84]. These data suggest that capillaries cluster in those cortical places where there are high densities of both astrocytes and spines. Mathematically, this means that the probability of finding a capillary at some location (or equivalently, fraction of capillary volume *c*) is proportional to probability of finding both an astrocyte (equal to volume fraction *g*) and a spine (equal to volume fraction *s*). The simplest form of such a probabilistic relationship is their product, i.e.

$$\begin{array}{c} c=gs. \end{array}$$ (19)

There is some additional empirical support for that “product formula” based on a laminar distribution of microvasculature and synapses both for rodents and for primates. For mouse somatosensory cortex, capillary fraction and astrocyte density correlate across cortical layers [31], but their relationship is clearly nonlinear, as local peaks in these two variables often do not exactly match (compare Fig. 1 in [31]). It seems that inclusion of spine density in that relationship should improve the correlation with changes in capillary fraction variability. Specifically, capillary fraction exhibits a peak in cortical somatosensory layer 1, which is however absent in the corresponding astrocyte density [31], but it correlates well with the fact that the density of asymmetric synapses (mostly spines) is the largest in the layer 1 of mouse somatosensory cortex [85].

For primate visual cortex, capillary length density is the largest in the middle layers 4 and 2/3, and the smallest in layers 1 and 5/6 [30, 86], which is similar to the laminar distribution of synaptic density, although with some fluctuations [87, 88]. Taken together, these data suggest that microvasculature is correlated with both synapses and astrocytes in the cortical gray matter.

Capillary fraction *c* in Eq (19) can be expressed in terms of spine parameters using Eq (17) for *g*, with the result

$$\begin{array}{c} c=\frac{as^{5/3}}{{\bar{u}}^{2/3}}. \end{array}$$ (20)

Note, that because *s* ∼ *g* ^3/2^, we have equivalently that *c* ∼ *g* ^5/2^, which indicates a strong nonlinear dependence between capillary and astrocyte volume fractions, and suggests that generally one can expect *c*/*g* ≪ 1.

#### The fitness functions

We consider three different classes of fitness functions. The first corresponds to the principle of neural “wire minimization” and the second to the proposed here “spine economical maximization”. The third class is a combination of the first two.

Wire minimization principle.

The most general form of the fitness function *F*_*w*_ (benefit-cost or Lagrange function) for wire minimization takes the form:

$$\begin{array}{c} F_{w}=\frac{rx+y}{{\bar{u}}^{\gamma_{1}}}+\lambda_{1}\;(x+y+s+g+c-1), \end{array}$$ (21)

where the exponent *γ*_1_ > 0 corresponds to specific characteristics of neuronal wire one wants to minimize (wire length, its surface area, its volume or conduction delays; see below). The free parameter *r* is a measure of the asymmetry between axons and dendrites. The parameter *λ*_1_ is the Lagrange multiplier associated with the volume normalization constraint: *x* + *y* + *s* + *g* + *c* = 1, i.e., fractional volumes of all considered cortical components must sum up to unity. The fitness function $F_{w}\left(x,y,\bar{u}\right)$ is the function of the three independent variables *x*, *y*, and $\bar{u}$, because the fractions *s*, *g*, and *c* depend on these variables (see Eqs (2), (17) and (20)).

The form of the benefit-cost function in Eq (21) can be justified by taking neural wire length minimization as an example. The other cases can be analyzed analogously. The total axonal volume in the cortex is $V_{a}=\left(\pi /4\right)L_{a}d_{a}^{2}$, where *L*_*a*_ is the total axon length in the cortex and *d*_*a*_ is the average axon diameter. Similarly, for the total dendrite volume we have $V_{d}=\left(\pi /4\right)L_{d}d_{d}^{2}$, where *L*_*d*_ is the total dendrite length and *d*_*d*_ is its diameter. Now, consider the fitness function *F*_*w*0_ as a density of combined lengths of axons and dendrites with some proportion coefficient *r*_0_:

$$\begin{array}{c} F_{w0}∼\frac{r_{0}L_{a}+L_{d}}{V}, \end{array}$$ (22)

where *V* is the volume of cortical gray matter. If we represent *L*_*a*_ and *L*_*d*_ in terms of *V*_*a*_ and *V*_*d*_, and denote the fractional volumes of axons and dendrites respectively as *x* = *V*_*a*_/*V* and *y* = *V*_*d*_/*V*, then we obtain that

$$\begin{array}{c} F_{w0}∼\frac{{\left(d_{d}/d_{a}\right)}^{2}r_{0}x+y}{d_{d}^{2}}. \end{array}$$ (23)

The empirical data indicate that average spine heads (postsynaptic density PSD) and dendrites have diameters of the same order of magnitude (fraction of micron), which do not seem to depend significantly on brain size [14]. This suggests that these two diameters can be mutually coupled, which implies that one can assume that $d_{d}^{2}∼{\bar{u}}^{\gamma_{1}}$, where the exponent *γ*_1_ ≈ 2/3 (the bulk of the spine has a spherical shape). Moreover, if we denote *r* = *r*_0_(*d*_*d*_/*d*_*a*_)^2^, then we obtain Eq (21) for the full (with the Lagrange multiplier term) fitness function.

A similar analysis performed for wire surface minimization and wire volume minimization yields the same formula but with different *γ*_1_, respectively 1/3 and 0. For the case of conduction delays minimization, one can define a similar fitness function to *F*_*w*0_ in Eq (22) with the substitutions $L_{a}\;\mapsto \;L_{a}/\sqrt{d_{a}}$ and $L_{d}\;\mapsto \;L_{d}/\sqrt{d_{d}}$, which approximately correspond to temporal delays along axons and dendrites, as the conduction velocity is proportional to a square root of unmyelinated wire diameter [48]. Performing the analysis analogously, one obtains Eq (21) with *γ*_1_ = 5/6 and *r* = *r*_0_(*d*_*d*_/*d*_*a*_)^5/2^.

Spine economical maximization principle.

The most general form of the fitness function *F*_*s*_ for spine proportion economical maximization takes the form:

$$\begin{array}{c} F_{s}=\frac{s}{{\bar{u}}^{\gamma_{2}}}+\lambda_{2}\;(x+y+s+g+c-1), \end{array}$$ (24)

where the exponent *γ*_2_ > 0 characterizes the influence of spine size on the maximization process (*γ*_2_ here is generally numerically different than *γ*_1_ in Eq (21)), and the parameter *λ*_2_ is the Lagrange multiplier as before. This proposition can be justified as follows. The analysis of experimental data on the developing cerebral cortex in several mammals indicate that the ratio of cerebral metabolic rate CMR (the rate of glucose consumption per cortical volume) and synaptic density *ρ*_*s*_ is approximately conserved from birth until adulthood for a given region of the cortex, despite large variabilities in CMR and *ρ*_*s*_ [27]. This means that CMR/*ρ*_*s*_ ≈ const. Synaptic density is comprised mostly of the density of spines (synapses in the cerebral cortex are in 80–90% excitatory, most of which are axo-spinal [18], and this percentage does not depend on brain size; [14]), which is related to spine fractional volume *s* via the formula $s=\rho_{s}\bar{u}$. This implies that the ratio CMR$\bar{u}/s$ is roughly conserved during development. The product in the nominator can be identified as an average metabolic energy per spine, which should be proportional to spine volume at some power *γ*_2_ ≥ 0, i.e. CMR$\bar{u}∼{\bar{u}}^{\gamma_{2}}$, because spine energy is associated mainly with the activity of Na/K-ATP pumps located on spine surface area [21, 89]. Thus, we obtain that the ratio $s/{\bar{u}}^{\gamma_{2}}$ should be developmentally approximately constant for a given cortical area, which indicates that this ratio is probably important for cortical functioning. Consequently, it is assumed here that there has been an evolutionary pressure on increasing the proportion of spines in the cortex that would consume the least energy or take the smallest size (spines are energy demanding [25–27]). These considerations lead to the maximization of the benefit-cost function given by Eq (24).

Combined wire minimization and spine economical maximization as a meta principle.

The simplest fitness function *F* that generalizes both notions of wire minimization and spine maximization, and includes them simultaneously is a linear combination of their corresponding primary fitness functions *F*_*w*_ and *F*_*s*_. If part of *F* associated with *F*_*w*_ is to be minimized, and part of *F* related to *F*_*s*_ is to be maximized, then we must include *F*_*w*_ and *F*_*s*_ with opposite signs. Therefore, we define the meta fitness function *F*, which we want to minimize, as

$$\begin{array}{c} F=fF_{w}-\left(1-f\right)F_{s}, \end{array}$$ (25)

where *f* is the parameter controlling relative contributions of wire minimization and spine economy maximization Lagrangians, with the condition 0 ≤ *f* ≤ 1. As we gradually decrease *f* from 1 to 0, then the meta fitness function *F* changes its character form dominated by wire minimization to dominated by spine economy maximization. The case *f* = 1/2 corresponds to a symmetric situation when both wire min and spine max rules are equally important. Note that minimization of (−*F*_*s*_) is mathematically equivalent to maximization of *F*_*s*_. Eq (1) in the Results section is obtained after substitution of Eqs (21) and (24) for *F*_*w*_ and *F*_*s*_ in Eq (25).

#### Optimization of the fitness functions

Optimal fractional volumes of axons *x*, dendrites *y*, spines *s*, glia/astrocytes *g*, capillaries *c*, and optimal average spine volume $\bar{u}$ are found by taking partial derivatives of *F*, i.e., $\partial F/\partial x=\partial F/\partial y=\partial F/\partial \bar{u}=\partial F/\partial \lambda =0$. As a result we obtain a system of three basic equations (details are provided in Supp. Information S1 Text):

$$\begin{array}{c} \left(1-f\right)s\left(y-x\right)+f{\bar{u}}^{\gamma_{2}-\gamma_{1}}\;[xy\left(1-r\right)+\left(y-rx\right)\;[s+\frac{g}{3}(2+5s)]]=0, \end{array}$$ (26)

$$\begin{array}{c} {}[\left(1-f\right)s{\bar{u}}^{\gamma_{1}}+f{\bar{u}}^{\gamma_{2}}\;(s+\frac{2}{3}g+\frac{5}{3}c)]\;\frac{y\bar{u}}{P}\frac{\partial P}{\partial \bar{u}}=\\ \left(y+s+\frac{2}{3}g+\frac{5}{3}c)\;[\left(1-f\right)\gamma_{2}s{\bar{u}}^{\gamma_{1}}-f\gamma_{1}\left(y+rx\right){\bar{u}}^{\gamma_{2}}\right]\\ +\frac{2}{3}(g+c)\;[fy{\bar{u}}^{\gamma_{2}}-\left(1-f\right)s{\bar{u}}^{\gamma_{1}}], \end{array}$$ (27)

and

$$\begin{array}{c} x+y+s+g+c=1. \end{array}$$ (28)

In the case of pure wire minimization principle, i.e. when *f* = 1, the above system reduces to the following equations (with explicit dependencies of *s*, *g*, and *c* on *x*, *y*, and *P*:

$$\begin{array}{c} \left(rx-y\right)\;[P+\frac{a}{3}{\left(\frac{P^{2}}{xy{\bar{u}}^{2}}\right)}^{1/3}\left(2+5Pxy\right)]=1-r, \end{array}$$ (29)

$$\begin{array}{c} \frac{2}{3}aPx^{2/3}y\left(1+Pxy\right)-\gamma_{1}\left(rx+y\right)P^{1/3}\;[{\bar{u}}^{2/3}y^{1/3}\left(1+Px\right)+\frac{a}{3}{\left(Px\right)}^{2/3}\left(2+5Pxy\right)]\\ =\bar{u}\frac{\partial P}{\partial \bar{u}}\;[P^{1/3}{\bar{u}}^{2/3}xy^{4/3}+\frac{a}{3}x^{2/3}y\left(2+5Pxy\right)], \end{array}$$ (30)

and

$$\begin{array}{c} x+y+Pxy+\frac{a{\left(Pxy\right)}^{2/3}}{{\bar{u}}^{2/3}}+\frac{a{\left(Pxy\right)}^{5/3}}{{\bar{u}}^{2/3}}=1. \end{array}$$ (31)

In the case of pure spine economical maximization principle, i.e. when *f* = 0, the system of Eqs (26–28) reduces to two equations:

$$\begin{array}{c} {\bar{u}}^{2/3}\frac{\partial P}{\partial \bar{u}}=\frac{P}{\bar{u}}\;(\gamma_{2}{\bar{u}}^{2/3}\left(1+Px\right)+\frac{a}{3}P^{2/3}x^{1/3}\left[2\left(\gamma_{2}-1\right)+\left(5\gamma_{2}-2\right)Px^{2}\right]) \end{array}$$ (32)

and

$$\begin{array}{c} 2x+Px^{2}+\frac{a{\left(Px^{2}\right)}^{2/3}}{{\bar{u}}^{2/3}}+\frac{a{\left(Px^{2}\right)}^{5/3}}{{\bar{u}}^{2/3}}=1, \end{array}$$ (33)

since in this case *x* = *y*.

The above equations are solved numerically for each distribution of spine volumes, using standard techniques [90]. In the case of pure wire minimization, the optimal solution corresponds to a local minimum of *F*_*w*_, whereas for pure spine economy maximization the optimal solution is associated with a local maximum of *F*_*s*_ (see S1 Text). For a mixed case with 0 < *f* < 1, the optimal solution is a local minimum of *F* (S1 Text).

#### Comparison of the theoretical results to the data

The optimal theoretical fractional volumes were compared to the empirical fractional volumes in the cerebral cortex using two related measures of similarity: Euclidean distance (ED) and Mahalanobis distance (MD). The major difference between these two measures is in their treatment of variance in the data. ED measures a distance between theoretical points and mean values of data points ignoring their variance, whereas MD measures such a normalized distance including standard deviations of data points [91]. Thus MD is more general than ED. The best similarity with the data is achieved for minimal values of MD and ED.

ED distance between theoretical and experimental points is defined as:

$$\begin{array}{c} \text{ED}=\sqrt{\sum_{i=1}^{5}{\left(x_{i}-x_{ex,i}\right)}^{2}}, \end{array}$$ (34)

where *x*_*i*_ and *x*_*ex*,*i*_ are respectively theoretical and mean empirical values of fractional volumes of axons, dendrites, spines, glia/astrocytes, and capillaries in the cortex (experimental data correspond to the next-to-last line in Table 1).

MD distance between theoretical and experimental points is defined as [91]:

$$\begin{array}{c} \text{MD}=\sqrt{\sum_{i=1}^{5}{\left(\frac{x_{i}-x_{ex,i}}{sd_{ex,i}}\right)}^{2}}, \end{array}$$ (35)

where *x*_*i*_ and *x*_*ex*,*i*_ are the same as in Eq (34), and *sd*_*ex*,*i*_ denotes the standard deviation associated with each data point (the next-to-last line in Table 1). It is easy to see that MD defined above is simply a variance-normalized ED distance. In a special case when all standard deviations *sd*_*ex*,*i*_ are equal to unity, MD reduces to ED.

## Supporting Information

S1 Text This file contains Supporting Figure A and Supporting Tables A-E. It also provides some details of the derivations, and proofs of minimum for *F*_*w*_ and *F*, and proof of maximum for *F*_*s*_.
(PDF)
