# Supplementary material for: Cortical Composition Hierarchy Driven by Spine Proportion Economical Maximization or Wire Volume Minimization
Source: PLoS Comput Biol. 2015 Oct 5;11(10):e1004532. doi: 10.1371/journal.pcbi.1004532 (PMC4593638; doi:10.1371/journal.pcbi.1004532)
Supplement: S1 Text — It also provides some details of the derivations, and proofs of minimum for F w and F, and proof of maximum for F s. (PDF) [file pcbi.1004532.s001.pdf]

Supporting Information for:

Cortical composition hierarchy driven by spine proportion economical maximization or wire volume minimization

Jan Karbowski

Institute of Applied Mathematics and Mechanics,

University of Warsaw, ul. Banacha 2, 02-097 Warsaw, Poland;

Email: jkarbowski@mimuw.edu.pl

Supporting Figure

Figure A. (see below)

Euclidean distance ED for heavy-tailed distributions of spine sizes as a function of exponent γ_2 for spine economical maximization. (A) ED for Log-logistic distribution with different values of β. (B) ED for Log-normal distribution with different values of σ. In both panels $\theta = 0.321\ \mu\text{m}^3$.

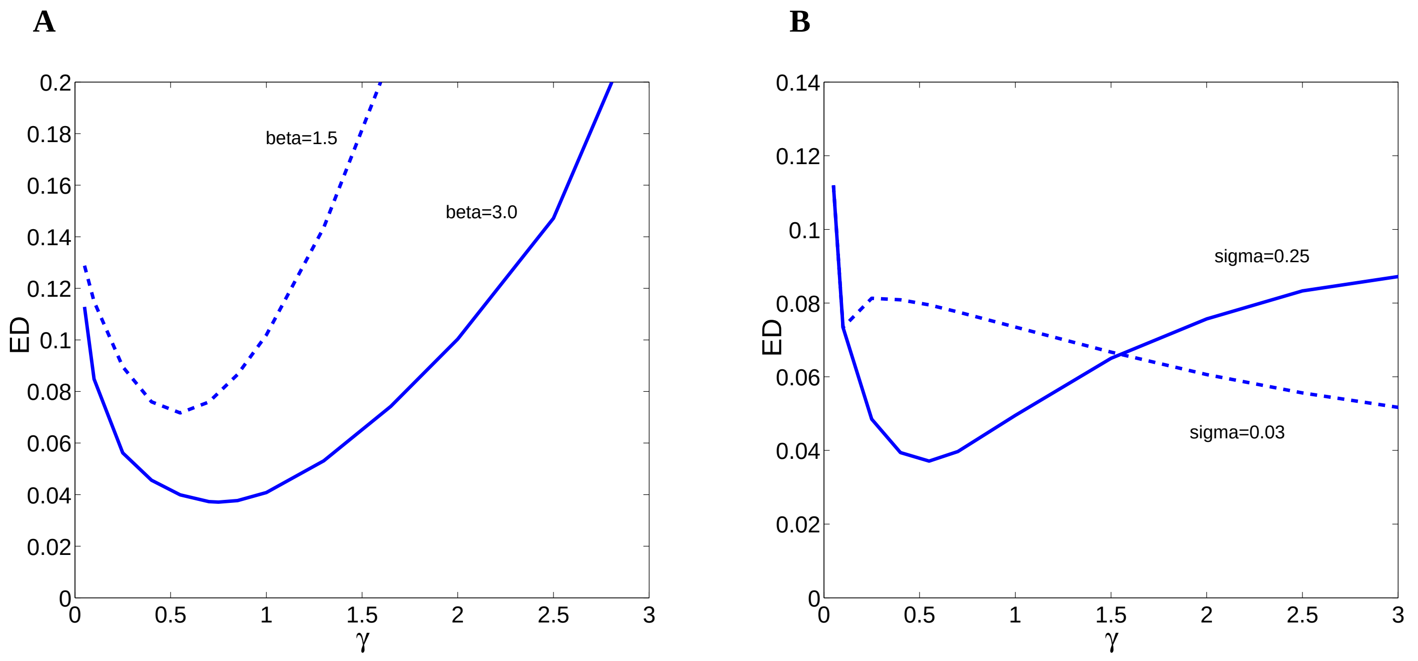

THEORETICAL MODELS

Below, the details of calculaions are provided for the three principles considered in the main text.

1 Wire minimization principle.

1.1 The system of basic equations for optimal solution.

Explicit form of the fitness function.

The explicit dependence of the fitness function F_w on the three parameters $x, y, \overline{u}$ is given as

$$F_w = \frac{rx + y}{\overline{u}^{\gamma_1}} + \lambda_1 \left(x + y + Pxy + \frac{a(Pxy)^{2/3}}{\overline{u}^{2/3}} + \frac{a(Pxy)^{5/3}}{\overline{u}^{2/3}} - 1 \right), \quad (1)$$

where the parameter $a = (3\pi^2/256)^{1/3} d_{as}^2 = 0.352\ \mu\text{m}^2$.

The basic optimal equations.

The optimal values of fractional volumes of axons and dendrites x, y, average spine volume $\overline{u}$, and the Lagrange multiplier λ_1 are found by differentiating the benefit-cost function F_w with respect to $x, y, \overline{u}$, and λ_1, and requiring that

$\partial F_w/\partial x = \partial F_w/\partial y = \partial F_w/\partial \overline{u} = \partial F_w/\partial \lambda_1 = 0$,

which corresponds to a critical point of F_w. Consequently, we obtain the following set of four nonlinear equations:

$$r + \lambda_1 \overline{u}^{\gamma_1} \left(1 + Py + \frac{a}{3} \left(\frac{P^2 y^2}{x \overline{u}^2} \right)^{1/3} [2 + 5Pxy] \right) = 0, \tag{2}$$

$$1 + \lambda_1 \overline{u}^{\gamma_1} \left(1 + Px + \frac{a}{3} \left(\frac{P^2 x^2}{y \overline{u}^2} \right)^{1/3} [2 + 5Pxy] \right) = 0, \tag{3}$$

$$\gamma_1 (rx + y) = \lambda_1 \overline{u}^{\gamma_1 + 1} \left[\frac{\partial P}{\partial \overline{u}} \left(xy + \frac{a(xy)^{2/3}}{3P^{1/3} \overline{u}^{2/3}} (2 + 5Pxy) \right) - \frac{2a(Pxy)^{2/3}}{3\overline{u}^{5/3}} (1 + Pxy) \right] \tag{4}$$

and

$$x + y + Pxy + \frac{a(Pxy)^{2/3}}{\overline{u}^{2/3}} + \frac{a(Pxy)^{5/3}}{\overline{u}^{2/3}} = 1. \tag{5}$$

Reduction of dimensionality in the system of basic equations.

Next, we show that we can decrease the number of basic equations from 4 to 3. First, we can get rid of λ_1, since it always appears in the first power. The parameter λ_1 can be determined from Eq. (3) and it reads:

$$\lambda_1 = -\frac{1}{\overline{u}^{\gamma_1}\left(1 + Px + \frac{a}{3}\left(\frac{P^2x^2}{\overline{u}^2 y}\right)^{1/3}[2 + 5Pxy]\right)}. \tag{6}$$

Next, we can insert λ_1 into Eqs. (2) and (4). As a result we obtain Eqs. (29) and (30) in the main text.

1.2 Proof of the local minimum for optimal solution related to wire minimization.

Let us introduce the following notation: $x_1 \equiv x$, $x_2 \equiv y$, $x_3 \equiv \overline{u}$. Then the function F_w (Eq. 1) can be rewritten as:

$$F_w = \frac{rx_1 + x_2}{x_3^{\gamma_1}} + \lambda_1 g(x_1, x_2, x_3), \tag{7}$$

where $g(x_1, x_2, x_3)$ denotes the constraint term present in Eq. (1). Let us define partial derivatives: $F_{ij} = \partial^2 F_w / \partial x_i \partial x_j$ and $g_i = \partial g / \partial x_i$, which are determined at the critical point represented by optimal values of x_1, x_2, x_3. Using these definitions we can construct a matrix called bordered Hessian for our constraint optimization problem as [2]:

$$\begin{bmatrix} 0 & g_1 & g_2 & g_3 \\ g_1 & F_{11} & F_{12} & F_{13} \\ g_2 & F_{21} & F_{22} & F_{23} \\ g_3 & F_{31} & F_{32} & F_{33} \end{bmatrix}$$

This is a symmetric matrix, i.e. $F_{ij} = F_{ji}$.

A sufficient condition for F_w to have a local minimum at the critical point represented by the optimal values x_1, x_2, x_3 is that two principal minors, i.e. determinants of the upper-left sub-matrices 3x3 (called D_1) and 4x4 (determinant of the entire bordered Hessian called D_2), have negative signs [2]. The explicit forms of these determinants are as follows:

$$D_1 = -g_1^2 F_{22} - g_2^2 F_{11} + g_1 g_2 (F_{12} + F_{21}) \tag{8}$$

and

$$\begin{aligned} D_2 = g_1^2(F_{23}^2 - F_{22}F_{33}) + g_2^2(F_{13}^2 - F_{11}F_{33}) \\ +g_3^2(F_{12}^2 - F_{11}F_{22}) + 2g_1g_2(F_{12}F_{33} - F_{13}F_{23}) \\ +2g_1g_3(F_{13}F_{22} - F_{12}F_{23}) + 2g_2g_3(F_{11}F_{23} - F_{12}F_{13}) \end{aligned} \tag{9}$$

Exact numerical values of the minors D_1 and D_2 are presented in Table A (below) together with the values of F_{ij}. These results indicate that indeed we have local minima at the critical points.

Table A: "Wire minimization" approach. The numerical values of the elements of the bordered Hessian and principal minors for each type of the spine volume distribution.

Spine size	θ	Bordered Hessian						Minors	
distribution		F_{11}	F_{12}	F_{22}	F_{13}	F_{23}	F_{33}	D_1	D_2
Exponential	0.100 (ED)	0.117	-0.905	0.162	-0.196	-0.230	2.476	-5.529	-13.69
	0.100 (MD)	0.107	-0.890	0.174	-0.185	-0.236	2.433	-5.449	-13.25
	0.321 (ED)	0.031	-0.748	0.041	-0.020	-0.023	0.026	-3.365	-0.087
	0.321 (MD)	0.036	-0.764	0.036	-0.022	-0.022	0.026	-3.433	-0.090
Gamma (n=1)	0.100 (ED)	0.110	-1.045	0.156	-0.191	-0.227	3.205	-7.020	-22.49
	0.100 (MD)	0.074	-0.967	0.216	-0.146	-0.249	2.919	-6.579	-19.19
	0.321 (ED)	0.033	-0.843	0.045	-0.026	-0.031	0.082	-4.148	-0.341
	0.321 (MD)	0.037	-0.856	0.041	-0.028	-0.030	0.084	-4.211	-0.352
Gamma (n=2)	0.100 (ED)	0.112	-1.118	0.156	-0.201	-0.238	4.440	-7.925	-35.19
	0.100 (MD)	0.063	-0.993	0.254	-0.134	-0.269	3.813	-7.196	-27.42
	0.321 (ED)	0.036	-0.891	0.047	-0.031	-0.036	0.155	-4.585	-0.712
	0.321 (MD)	0.038	-0.901	0.045	-0.033	-0.035	0.157	-4.632	-0.727
Rayleigh	0.100 (ED)	0.117	-1.121	0.171	-0.219	-0.265	5.876	-8.101	-47.60
	0.100 (MD)	0.063	-0.987	0.288	-0.141	-0.301	4.979	-7.327	-36.45
	0.321 (ED)	0.040	-0.890	0.052	-0.036	-0.042	0.199	-4.608	-0.918
	0.321 (MD)	0.042	-0.899	0.050	-0.038	-0.041	0.201	-4.655	-0.938
Log-logistic	0.100 (ED)	0.060	-0.856	0.080	-0.065	-0.075	0.243	-4.482	-1.088
	0.100 (MD)	0.062	-0.860	0.078	-0.067	-0.075	0.244	-4.505	-1.099
	0.321 (ED)	0.037	-0.936	0.048	-0.035	-0.040	0.310	-5.012	-1.555
	0.321 (MD)	0.039	-0.967	0.057	-0.042	-0.050	0.594	-5.394	-3.205
Log-normal	0.100 (ED)	0.073	-0.696	0.095	-0.079	-0.090	1.323	-3.290	-4.354
	0.100 (MD)	0.089	-0.791	0.113	-0.114	-0.129	3.283	-4.140	-13.59
	0.321 (ED)	0.045	-0.838	0.061	-0.042	-0.049	0.985	-4.206	-4.141
	0.321 (MD)	0.050	-0.925	0.069	-0.055	-0.064	2.458	-5.061	-12.44

For each value of θ there are two values of Hessian matrix and Minors corresponding to minimal Euclidean (ED) and Mahalanobis (MD) distances.

2 Spine economical maximization principle.

2.1 The system of basic equations for optimal solution.

Explicit form of the fitness function.

The explicit dependence of the benefit-cost function F_s on the three parameters $x, y, \overline{u}$ is given as

$$F_s = \frac{Pxy}{\overline{u}^{\gamma_2}} + \lambda_2 \left(x + y + Pxy + \frac{a(Pxy)^{2/3}}{\overline{u}^{2/3}} + \frac{a(Pxy)^{5/3}}{\overline{u}^{2/3}} - 1 \right). \quad (10)$$

The basic optimal equations.

The optimal values of x, y, $\overline{u}$, and λ_2 are found by differentiating the benefit-cost function F_s (Eq. 10) with respect to $x, y, \overline{u}$, and λ_2, and requiring that appropriate derivatives are zero. As a result, we obtain the following set of four nonlinear equations:

$$Py + \lambda_2 \overline{u}^{\gamma_2} \left(1 + Py + \frac{a}{3} \left(\frac{P^2 y^2}{x \overline{u}^2} \right)^{1/3} [2 + 5Pxy] \right) = 0, \quad (11)$$

$$Px + \lambda_2 \overline{u}^{\gamma_2} \left(1 + Px + \frac{a}{3} \left(\frac{P^2 x^2}{y \overline{u}^2} \right)^{1/3} [2 + 5Pxy] \right) = 0, \quad (12)$$

$$\left[(\frac{1}{\overline{u}^{\gamma_2}} + \lambda_2)(xy)^{1/3} + \frac{a\lambda_2(2+5Pxy)}{3(P\overline{u}^2)^{1/3}}\right]\frac{\partial P}{\partial \overline{u}} = \left[\frac{\gamma_2 (xy)^{1/3}}{\overline{u}^{\gamma_2+1}} + \frac{2a\lambda_2(1+Pxy)}{3(P\overline{u}^5)^{1/3}}\right] P, \quad (13)$$

and

$$x + y + Pxy + \frac{a(Pxy)^{2/3}}{\overline{u}^{2/3}} + \frac{a(Pxy)^{5/3}}{\overline{u}^{2/3}} = 1. \quad (14)$$

Note that from Eqs. (11) and (12) it follows that λ_2 must be negative, since all other terms on the left hand side are positive. This observation is used below for determination of the type of extremum.

Proof that $x = y$.

First, we show that for optimal x and y we have $x = y$. To do this, we subtract Eqs. (11) and (12). As a result we get:

$$(y - x)\left[P(1 + \lambda_2\overline{u}^{\gamma_2}) + \lambda_2\overline{u}^{\gamma_2}\frac{a}{3}\left(\frac{P^2}{xy\overline{u}^2}\right)^{1/3}(2 + 5Pxy)\right] = 0, \quad (15)$$

where the expression in the [...] bracket is equal either to $-\lambda_2\overline{u}^{\gamma_2}/y$ (from Eq. 11) or to $-\lambda_2\overline{u}^{\gamma_2}/x$ (from Eq. 12). Thus, Eq. (15) is equivalent to the following equation:

$$(y-x)\frac{\lambda_2\overline{u}^{\gamma_2}}{y}=0, \tag{16}$$

which implies that for nonzero λ_2 and $\overline{u}$ we must have $x=y$. (The benefit-cost function F_s is defined only for $\overline{u}>0$, see Eq. 10). If however, $\lambda_2=0$, then from Eqs. (11) and (12) we get that $Px=Py=0$. The case $P=0$ implies $\overline{u}=0$ (see eqs relating P and $\overline{u}$ in the Methods), which however is forbidden. Thus $P\neq 0$, and in this case we must have $x=y=0$, i.e. x and y are still equal to each other.

Reduction of dimensionality in the system of basic equations.

Next, we show that we can decrease the number of basic equations. Because $x=y$, we can reduce the system of 4 equations to the system of 3 equations with unknowns $x,\overline{u},\lambda_2$ (Eqs. 11 and 12 are in fact the same equation). Moreover, we can get rid of λ_2, since it always appears in the first power, which additionally allows us to reduce the system dimensionality to 2. The parameter λ_2 can be determined from Eq. (11) (with the substitution $y=x$) and it reads:

$$\lambda_2=-\frac{Px}{\overline{u}^{\gamma_2}\left(1+Px+\frac{a}{3}\left(\frac{P^2x}{\overline{u}^2}\right)^{1/3}[2+5Px^2]\right)}. \tag{17}$$

Next, we can insert λ_2 into Eq. (13). After this procedure Eq.(13) becomes

$$\overline{u}^{2/3}\frac{\partial P}{\partial\overline{u}}=\frac{P}{\overline{u}}\left(\gamma_2\overline{u}^{2/3}(1+Px)+\frac{a}{3}P^{2/3}x^{1/3}[2(\gamma_2-1)+(5\gamma_2-2)Px^2]\right) \tag{18}$$

and Eq. (14) after the substitution $y = x$ becomes

$$2x + Px^2 + \frac{aP^{2/3}x^{4/3}}{\overline{u}^{2/3}} + \frac{aP^{5/3}x^{10/3}}{\overline{u}^{2/3}} = 1. \tag{19}$$

The derivatives of P with respect to $\overline{u}$ have different forms depending on the type of density probability of spine volumes $H(u)$ (see the main text).

Eqs. (18) and (19) constitute the reduced system of basic equations, which is used for computations of two independent variables x and $\overline{u}$. This two-dimensional system can be solved by a handful of numerical techniques (e.g. [1]).

2.2 Proof of the local maximum for optimal solution related to spine economy.

As before, let us introduce the following notation: $x_1 \equiv x$, $x_2 \equiv y$, $x_3 \equiv \overline{u}$. Then the fitness function F_s (Eq. 10) can be rewritten as:

$$F_s = \frac{Px_1x_2}{x_3^{\gamma_2}} + \lambda_2 g(x_1, x_2, x_3), \tag{20}$$

where the probability P is a function of x_3, and $g(x_1, x_2, x_3)$ denotes the constraint term present in Eq. (10). Let us define partial derivatives: $F_{ij} = \partial^2 F_s/\partial x_i \partial x_j$ and $g_i = \partial g/\partial x_i$,

which are determined at the critical point represented by optimal values of x_1, x_2, x_3. Using these definitions we can construct a matrix called bordered Hessian for our constraint optimization problem as [2]:

$$\begin{bmatrix} 0 & g_1 & g_2 & g_3 \\ g_1 & F_{11} & F_{12} & F_{13} \\ g_2 & F_{21} & F_{22} & F_{23} \\ g_3 & F_{31} & F_{32} & F_{33} \end{bmatrix}$$

This particular matrix has a high degree of symmetry, since: $g_1 = g_2$, $F_{ij} = F_{ji}$, and additionally $F_{11} = F_{22}$, $F_{13} = F_{23}$.

A sufficient condition for F_s to have a local maximum at the critical point represented by the optimal values x_1, x_2, x_3 is that two principal minors, i.e. determinants of the upper-left sub-matrices 3x3 (called D_1) and 4x4 (determinant of the entire bordered Hessian called D_2), alternate in sign. Specifically, the principal minors must have respectively positive (D_1) and negative (D_2) signs [2]. Using the high symmetry in the Hessian matrix, the explicit forms of these determinants are as follows:

$$D_1 = 2g_1^2(F_{12} - F_{11}) \tag{21}$$

and

$$D_2 = g_1^2(F_{12} - F_{11})\left[2F_{33} - 4\epsilon F_{13} + \epsilon^2(F_{12} + F_{11})\right], \tag{22}$$

where $\epsilon \equiv g_3/g_1$. It is relatively easy to show that $g_1 \geq 1$, and the expression for ϵ reads

$$\epsilon = \frac{x}{P}\left(\frac{\partial P}{\partial \overline{u}} - \frac{\gamma_2 P}{\overline{u}}\right). \qquad (23)$$

In general for all considered distributions of spine volume, the numerical value of ϵ is very small at the critical point, i.e. $|\epsilon| \ll 1$. Typical values of F_{ij} are in the range $(-1.7, 1)$. Thus, approximately the sign of D_2 is determined by the sign of the product $F_{33}(F_{12} - F_{11})$, since other terms in Eq. (22) are much smaller and thus can be neglected. Of these two factors, F_{33} is always negative (which comes from a numerical calculation) and $(F_{12} - F_{11}) = -\lambda_2/x$ is always positive ($\lambda_2 < 0$). This implies that D_2 is negative, and D_1 is positive, which is sufficient for the benefit-cost function F_s (Eq. 10) to have maximum. Exact numerical values of the rescaled minors D_1/g_1^2 and D_2/g_1^2 are presented in Table B (below) together with the values of ϵ and F_{ij}.

Table B: "Spine economical maximization". The numerical values of the elements of the bordered Hessian and principal minors for each type of the spine volume distribution.

Spine size distribution	θ	Bordered Hessian					Minors	
		ϵ	F_{11}	F_{12}	F_{13}	F_{33}	D_1/g_1^2	D_2/g_1^2
Exponential	0.100 (ED)	-0.053	0.017	0.621	-0.018	-0.059	1.207	-0.072
	0.100 (MD)	-0.053	0.017	0.621	-0.018	-0.059	1.207	-0.072
	0.321 (ED)	0.024	0.016	0.540	-0.010	-0.145	1.048	-0.151
	0.321 (MD)	0.014	0.014	0.526	-0.009	-0.098	1.023	-0.100
Gamma (n=1)	0.100 (ED)	-0.072	0.017	0.657	-0.020	-0.055	1.279	-0.072
	0.100 (MD)	-0.072	0.017	0.657	-0.020	-0.055	1.279	-0.072
	0.321 (ED)	0.061	0.022	0.664	-0.014	-0.397	1.284	-0.506
	0.321 (MD)	0.017	0.016	0.606	-0.012	-0.165	1.180	-0.194
Gamma (n=2)	0.100 (ED)	-0.062	0.013	0.645	-0.014	-0.024	1.263	-0.031
	0.100 (MD)	-0.062	0.013	0.645	-0.014	-0.024	1.263	-0.031
	0.321 (ED)	0.085	0.025	0.739	-0.016	-0.653	1.427	-0.924
	0.321 (MD)	0.033	0.020	0.669	-0.015	-0.315	1.298	-0.408
Rayleigh	0.100 (ED)	-0.098	0.020	0.677	-0.027	-0.072	1.314	-0.097
	0.100 (MD)	-0.058	0.013	0.641	-0.013	-0.021	1.256	-0.028
	0.321 (ED)	0.067	0.025	0.734	-0.018	-0.654	1.418	-0.921
	0.321 (MD)	0.015	0.019	0.662	-0.016	-0.283	1.285	-0.362
Log-logistic	0.100 (ED)	-0.016	0.022	0.654	-0.022	-0.189	1.264	-0.240
	0.100 (MD)	-0.016	0.022	0.654	-0.022	-0.189	1.264	-0.240
	0.321 (ED)	0.124	0.026	0.778	-0.014	-0.895	1.505	-1.332
	0.321 (MD)	0.075	0.025	0.784	-0.019	-0.949	1.518	-1.432
Log-normal	0.100 (ED)	-0.074	0.021	0.582	-0.025	-0.247	1.121	-0.279
	0.100 (MD)	-0.074	0.017	0.616	-0.019	-0.076	1.199	-0.092
	0.321 (ED)	0.044	0.023	0.683	-0.018	-1.737	1.321	-2.292
	0.321 (MD)	-0.030	0.021	0.666	-0.022	-0.797	1.290	-1.029

For each value of θ there are two values of Hessian matrix and Minors corresponding to minimal Euclidean (ED) and Mahalanobis (MD) distances.

3 Combined “wire minimization” and “spine economy maximization” principle.

3.1 The system of basic equations for optimal solution.

The optimal values of x, y, $\overline{u}$, and λ are found by differentiating the meta fitness function F (Eq. 1 in the main text) with respect to $x, y, \overline{u}$, and λ, and requiring that appropriate derivatives are zero. As a result, we obtain the following set of four nonlinear equations:

$$\frac{frx}{\overline{u}^{\gamma_1}} - \frac{(1-f)s}{\overline{u}^{\gamma_2}} + \lambda\left(x + s + \frac{2}{3}g + \frac{5}{3}c\right) = 0, \tag{24}$$

$$\frac{fy}{\overline{u}^{\gamma_1}} - \frac{(1-f)s}{\overline{u}^{\gamma_2}} + \lambda\left(y + s + \frac{2}{3}g + \frac{5}{3}c\right) = 0, \tag{25}$$

$$\left(\left[\lambda - \frac{(1-f)}{\overline{u}^{\gamma_2}}\right]s + \frac{\lambda g}{3}(2+5s)\right)\frac{\overline{u}}{P}\frac{\partial P}{\partial \overline{u}} - \frac{2}{3}\lambda g(1+s) = \frac{\gamma_1 f}{\overline{u}^{\gamma_1}}(rx+y) - \frac{\gamma_2 s(1-f)}{\overline{u}^{\gamma_2}} \tag{26}$$

and

$$x + y + s + g + c = 1. \tag{27}$$

Reduction of dimensionality in the system of basic equations.

As before we can reduce the number of equations from 4 to 3. For this purpose, we determine λ from Eq. (25):

$$\lambda = \frac{(1-f)\frac{s}{\overline{u}^{\gamma_2}} - f\frac{y}{\overline{u}^{\gamma_1}}}{y + s + \frac{2}{3}g + \frac{5}{3}c} \tag{28}$$

and next, we insert this equation into Eqs. (24) and (26). As a result, we get Eqs. (26-27) in the main text.

3.2 Proof of the local maximum for optimal solution related to spine economy.

The bordered Hessian matrix can be determined similarly as in the previous two cases. A sufficient condition for the meta fitness function F (Eq. 1 in the main text) to have a local minimum is that two principal minors are negative. Exact numerical values of the minors and elements of the bordered Hessian are displayed in Tables C-E, for different mixing ratio f in F. These tables correspond to Tables 4-6 in the main text.

Table C: Combined "Wire min + spine max" approach for $f = 0.1$.

Principle type/	Bordered Hessian						Minors	
spine distr.	F_{11}	F_{12}	F_{22}	F_{13}	F_{23}	F_{33}	D_1	D_2
**wire length min + spine max**								
Exponential	-0.010	-0.694	-0.010	0.141	0.141	0.163	-2.870	-0.455
Gamma (n=1)	-0.013	-0.809	-0.014	0.154	0.153	0.324	-3.664	-1.154
Gamma (n=2)	-0.015	-0.895	-0.017	0.172	0.168	0.540	-4.228	-2.219
Rayleigh	-0.015	-0.898	-0.018	0.177	0.171	0.535	-4.289	-2.252
Log-logistic	-0.017	-0.963	-0.022	0.173	0.156	1.056	-4.867	-5.045
Log-normal	-0.019	-1.000	-0.022	0.228	0.222	2.370	-5.103	-12.13
**wire surface min + spine max**								
Exponential	-0.008	-0.598	-0.009	0.058	0.058	0.104	-2.488	-0.254
Gamma (n=1)	-0.011	-0.707	-0.012	0.068	0.067	0.230	-3.211	-0.726
Gamma (n=2)	-0.013	-0.764	-0.015	0.073	0.070	0.368	-3.626	-1.316
Rayleigh	-0.013	-0.760	-0.016	0.074	0.071	0.349	-3.645	-1.262
Log-logistic	-0.015	-0.843	-0.020	0.078	0.071	0.822	-4.264	-3.472
Log-normal	-0.023	-0.988	-0.030	0.112	0.100	6.943	-4.898	-33.89
**wire volume min + spine max**								
Exponential	-0.008	-0.545	-0.008	0.005	0.005	0.090	-2.267	-0.204
Gamma (n=1)	-0.011	-0.637	-0.012	0.008	0.008	0.209	-2.886	-0.601
Gamma (n=2)	-0.012	-0.681	-0.014	0.010	0.010	0.296	-3.239	-0.955
Rayleigh	-0.012	-0.674	-0.014	0.010	0.011	0.271	-3.240	-0.878
Log-logistic	-0.014	-0.759	-0.019	0.013	0.015	0.734	-3.833	-2.804
Log-normal	-0.015	-0.726	-0.021	0.016	0.019	1.938	-3.632	-7.038
**delays min + spine max**								
Exponential	-0.011	-0.751	-0.011	0.195	0.195	0.202	-3.102	-0.603
Gamma (n=1)	-0.014	-0.888	-0.015	0.220	0.219	0.411	-4.012	-1.589
Gamma (n=2)	-0.015	-0.941	-0.018	0.224	0.219	0.562	-4.461	-2.433
Rayleigh	-0.018	-1.004	-0.020	0.258	0.253	0.712	-4.778	-3.313
Log-logistic	-0.019	-1.076	-0.025	0.250	0.231	1.389	-5.407	-7.316
Log-normal	-0.022	-1.127	-0.026	0.326	0.316	3.596	-5.570	-19.96

Table D: Combined "Wire min + spine max" approach for $f = 0.5$.

Principle type/ spine distr.	Bordered Hessian F_{11}	F_{12}	F_{22}	F_{13}	F_{23}	F_{33}	Minors D_1	D_2
**wire length min + spine max**								
Exponential	-0.432	-16.79	-0.432	9.602	9.604	858.9	-43.24	-35899.2
Gamma (n=1)	-0.132	-6.323	-0.126	4.242	4.230	85.09	-19.83	-1542.5
Gamma (n=2)	-0.055	-4.065	-0.051	2.668	2.612	24.29	-15.13	-320.69
Rayleigh	-0.054	-4.001	-0.050	2.546	2.491	27.10	-15.66	-378.75
Log-logistic	-0.017	-2.778	-0.021	1.360	1.459	11.86	-13.58	-146.30
Log-normal	0.008	-1.679	0.010	0.871	1.000	1.299	-8.369	-10.948
**wire surface min + spine max**								
Exponential	-0.017	-2.345	-0.017	1.280	1.280	12.93	-7.794	-91.96
Gamma (n=1)	0.008	-1.536	0.008	0.583	0.583	1.576	-6.470	-8.808
Gamma (n=2)	0.012	-1.206	0.012	0.353	0.353	0.231	-5.844	-1.155
Rayleigh	0.010	-1.320	0.011	0.402	0.433	0.645	-6.353	-3.725
Log-logistic	0.005	-1.377	0.008	0.344	0.424	1.764	-7.203	-12.18
Log-normal	0.010	-1.317	0.013	0.384	0.448	3.027	-6.831	-20.67
**wire volume min + spine max**								
Exponential	0.017	-0.639	0.017	-0.011	-0.011	0.098	-2.754	-0.272
Gamma (n=1)	0.014	-0.733	0.016	-0.010	-0.011	0.208	-3.451	-0.720
Gamma (n=2)	0.013	-0.786	0.016	-0.011	-0.012	0.308	-3.877	-1.196
Rayleigh	0.014	-0.788	0.016	-0.012	-0.013	0.336	-3.921	-1.320
Log-logistic	0.011	-0.872	0.014	-0.009	-0.011	0.837	-4.561	-3.817
Log-normal	0.014	-0.810	0.019	-0.016	-0.018	1.980	-4.282	-8.477
**delays min + spine max**								
Exponential	-1.173	-47.71	-1.174	27.45	27.48	4239.5	-115.2	$-4.7\cdot10^5$
Gamma (n=1)	-0.290	-11.87	-0.290	8.223	8.223	280.65	-34.14	$-8.9\cdot10^3$
Gamma (n=2)	-0.142	-7.007	-0.142	5.057	5.057	82.31	-23.48	$-1.7\cdot10^3$
Rayleigh	-0.133	-6.587	-0.133	4.521	4.521	83.58	-23.60	$-1.8\cdot10^3$
Log-logistic	-0.017	-3.069	-0.022	1.785	1.994	12.03	-15.63	-170.03
Log-normal	-0.022	-3.329	-0.031	2.025	2.332	70.05	-16.83	-1156.2

Table E: Combined “Wire min + spine max” approach for $f = 0.9$.

Principle type/ spine distr.	Bordered Hessian						Minors	
	F_{11}	F_{12}	F_{22}	F_{13}	F_{23}	F_{33}	D_1	D_2
**wire length min + spine max**								
Exponential	-2$*10^{-7}$	-0.027	-2$*10^{-7}$	$3*10^{-8}$	$3*10^{-8}$	$7*10^{-13}$	-0.107	-8$*10^{-14}$
Gamma (n=1)	-28.19	-3134.4	-28.19	-854.0	-854.0	$8*10^{5}$	-6590.6	-5$*10^{9}$
Gamma (n=2)	-8.533	-666.9	-8.533	-169.3	-169.3	10^{5}	-1440.5	-10^{8}
Rayleigh	-6.136	-283.8	-6.136	-87.28	-87.28	$4*10^{4}$	-660.35	-2$*10^{7}$
Log-logistic	-2$*10^{-7}$	-0.027	-2$*10^{-7}$	$3*10^{-8}$	$3*10^{-8}$	$7*10^{-13}$	-0.107	-8$*10^{-14}$
Log-normal	-0.677	-20.66	-0.944	-1.887	-4.444	17389.8	-100.47	-2$*10^{6}$
**wire surface min + spine max**								
Exponential	-11.59	-1045.9	-11.59	-397.0	-397.0	$4*10^{5}$	-2222.0	-8$*10^{8}$
Gamma (n=1)	-1.608	-94.90	-1.608	-23.57	-23.57	12275.2	-211.3	-2$*10^{6}$
Gamma (n=2)	-0.606	-29.01	-0.606	-3.631	-3.631	2166.6	-68.41	-10^{5}
Rayleigh	-0.528	-20.09	-0.528	-2.217	-2.217	1437.8	-51.82	-7$*10^{4}$
Log-logistic	-0.084	-5.240	-0.118	0.747	0.745	329.89	-26.11	-8$*10^{3}$
Log-normal	-0.013	-3.494	-0.017	1.029	1.174	477.03	-17.12	-8$*10^{3}$
**wire volume min + spine max**								
Exponential	0.043	-0.725	0.043	-0.028	-0.028	0.079	-3.220	-0.257
Gamma (n=1)	0.042	-0.840	0.042	-0.031	-0.031	0.177	-4.074	-0.724
Gamma (n=2)	0.036	-0.878	0.047	-0.030	-0.034	0.284	-4.456	-1.273
Rayleigh	0.038	-0.879	0.049	-0.034	-0.038	0.325	-4.501	-1.470
Log-logistic	0.038	-0.986	0.049	-0.032	-0.036	0.948	-5.277	-5.015
Log-normal	0.044	-0.913	0.057	-0.046	-0.052	2.645	-4.907	-12.99
**delays min + spine max**								
Exponential	-2$*10^{-6}$	-0.034	-2$*10^{-6}$	$5*10^{-7}$	$3*10^{-7}$	10^{-10}	-0.135	-2$*10^{-11}$
Gamma (n=1)	-2$*10^{-6}$	-0.034	-2$*10^{-6}$	$5*10^{-7}$	$3*10^{-7}$	10^{-10}	-0.135	-2*10^{-11}
Gamma (n=2)	-20.49	-1961.9	-20.49	-488.7	-488.7	$3*10^{5}$	-4169.7	-2$*10^{9}$
Rayleigh	-16.33	-865.41	-16.33	-287.2	-287.2	10^{5}	-1961.9	-3$*10^{8}$
Log-logistic	-2$*10^{-6}$	-0.034	-2$*10^{-6}$	$5*10^{-7}$	$3*10^{-7}$	10^{-10}	-0.135	-2$*10^{-11}$
Log-normal	-0.746	-23.22	-1.035	-0.794	-3.932	19583.9	-112.99	-2$*10^{6}$

References

[1] Press WH, Teukolsky SA, Vetterling WT, Flannery BP (1992) *Numerical Recipes in Fortran*. Cambridge: Cambridge Univ. Press.

[2] Neudecker H, Magnus JR (1988) *Matrix Differential Calculus with Applications in Statistics and Econometrics*. New York: Wiley.
